# Model‐Based Prediction of Clinically Relevant Thrombocytopenia after Allogeneic Hematopoietic Stem Cell Transplantation

**DOI:** 10.1002/cpt.3580

**Published:** 2025-02-06

**Authors:** Katharina M. Götz, Amin T. Turki, Katharina Och, Dominik Selzer, Christian Brossette, Norbert Graf, Jochen Rauch, Stefan Theobald, Yvonne Braun, Kerstin Rohm, Gabriele Weiler, Simeon Rüdesheim, Matthias Schwab, Lisa Eisenberg, Nico Pfeifer, Stephan Kiefer, Ulf Schwarz, Claudia Riede, Sigrun Smola, Dietrich W. Beelen, Dominic Kaddu‐Mulindwa, Jürgen Rissland, Jörg Bittenbring, Thorsten Lehr

**Affiliations:** ^1^ Department of Clinical Pharmacy Saarland University Saarbrücken Germany; ^2^ Department of Hematology and Stem Cell Transplantation University Hospital Essen Essen Germany; ^3^ Department of Hematology and Oncology, Marienhospital University Hospital Ruhr‐University Bochum Bochum Germany; ^4^ Department of Pediatric Oncology and Hematology Saarland University Homburg Germany; ^5^ Department of Biomedical Data & Bioethics Fraunhofer Institute for Biomedical Engineering (IBMT) Sulzbach Germany; ^6^ Dr. Margarete Fischer‐Bosch‐Institute of Clinical Pharmacology Stuttgart Germany; ^7^ Department of Clinical Pharmacology, Pharmacy and Biochemistry University of Tübingen Tübingen Germany; ^8^ Cluster of Excellence iFIT (EXC2180) “Image‐guided and Functionally Instructed Tumor Therapies” University of Tübingen Tübingen Germany; ^9^ Department of Computer Science University of Tübingen Tübingen Germany; ^10^ Institute for Formal Ontology and Medical Information Science (IFOMIS) Saarland University Saarbrücken Germany; ^11^ Averbis GmbH Freiburg Germany; ^12^ Institute of Virology Saarland University Medical Center Homburg Germany; ^13^ Department of Internal Medicine 1 University Hospital Saarland Homburg Germany

## Abstract

Platelet reconstitution after allogeneic hematopoietic cell transplantation (allo‐HCT) is heterogeneous and influenced by various patient‐ and transplantation‐related factors, associated with poor prognoses for poor graft function (PGF) and isolated thrombocytopenia. Tailored interventions could improve the outcome of patients with PGF and post‐HCT thrombocytopenia. To provide individual predictions of 180‐day platelet counts from early phase data, we developed a model of long‐term platelet reconstitution after allo‐HCT. A large cohort (*n* = 1949) of adult patients undergoing their first allo‐HCT was included. Real‐world data from 1,048 retrospective patients were used for non‐linear mixed‐effects model development. Bayesian forecasting was used to predict platelet–time profiles for 518 retrospective and 383 prospective patients during internal and external model validation, respectively. Thrombocytopenia was defined as mean platelet count < 75 × 10^9^/L, derived from the last 12 platelet measurements within the first 180 days post‐HCT. Thrombocytopenia affected 37% of all patients and was associated with significantly reduced overall survival (*P*‐value < 0.0001). On days +7, +14, +21, and +28, the developed model achieved areas under the receiver‐operating characteristic of ≥ 0.68, ≥ 0.75, ≥ 0.78, and 0.81 for the prediction of post‐HCT thrombocytopenia, respectively, with anti‐thymocyte globulin, donor relation, and total protein measurements representing prognostic markers for post‐HCT platelet kinetics. A publicly accessible web‐based demonstrator of the model was established (https://hsct.precisiondosing.de). In summary, the developed model predicts individual platelet counts from day +28 post‐HCT adequately, utilizing internal and external datasets. The web‐based demonstrator provides a basis to implement model‐based predictions in clinical practice and to confirm these findings in future clinical studies.

**Study Highlights**

**WHAT IS THE CURRENT KNOWLEDGE ON THE TOPIC?**

Thrombocytopenia after allogeneic hematopoietic cell transplantation (allo‐HCT) is a critical concern, often linked to poor prognoses.

**WHAT QUESTION DID THIS STUDY ADDRESS?**

This study aimed at the development of a model for the early prediction of long‐term platelet reconstitution after allo‐HCT and to establish methods that provide access to model‐based predictions in clinical practice.

**WHAT DOES THIS STUDY ADD TO OUR KNOWLEDGE?**

On days +7, +14, +21 and +28 post‐HCT, the developed model of platelet reconstitution after allo‐HCT predicts individual platelet counts for up to 180 days adequately. Transplantation characteristics (anti‐thymocyte globulin, donor relation) and clinical data (serum total protein, early platelet counts) were the key determinants for post‐HCT platelet kinetics. A web‐based demonstrator of the model was hosted at https://hsct.precisiondosing.de.

**HOW MIGHT THIS CHANGE CLINICAL PHARMACOLOGY OR TRANSLATIONAL SCIENCE?**

The early identification of patients at risk for thrombocytopenia after allo‐HCT could support clinical decision‐making, such as timely donor stem cell boosts. The web‐based model demonstrator provides a basis to confirm these findings in future clinical studies.

Allogeneic hematopoietic cell transplantation (allo‐HCT) is a potentially curative treatment for different hematological diseases, but it can lead to severe complications such as graft‐versus‐host disease (GvHD) and the reconstitution of the marrow may be impaired. Poor graft function (PGF), characterized by low levels of neutrophils, platelets, or hemoglobin, is associated with a significantly decreased 1‐year overall survival (OS) after allo‐HCT.^1^, ^2^ Isolated thrombocytopenia, occurring early and late after allo‐HCT, significantly worsens OS.^3^, ^4^, ^5^, ^6^, ^7^, ^8^ While platelet counts at day +60 have been found to be associated with infectious events and non‐relapse mortality,^9^ insufficient platelet counts at day +100 have been identified as a predictor of mortality after allo‐HCT.^10^ To support the clinical decision for timely interventions, such as administration of thrombopoietin receptor agonists or donor stem cell boosts, personalized and precise predictions of long‐term platelet counts are required for improving allo‐HCT care.

Known risk factors for PGF are underlying disease (e.g., myelodysplastic syndrome vs. leukemia), infusion with low dose CD34+ cells, reactivation of Epstein–Barr virus and cytomegalovirus, high serum ferritin levels, and splenomegaly.^1^, ^2^, ^11^ Other risk factors for prolonged thrombocytopenia can be related to transplantation characteristics (underlying high‐risk disease, human leukocyte antigen (HLA)‐mismatched transplantation, myeloablative conditioning treatment, graft source other than sibling donor, AB0 major mismatch, recipient cytomegalovirus‐positive serostatus) or to complications after allo‐HCT (pulmonary infection within the first 100 days after HCT, grades II–IV acute GvHD).^4^, ^5^, ^12^ In addition, impairment of the bone marrow vascular microenvironment contributes to both PGF and prolonged thrombocytopenia.^1^, ^13^, ^14^

Since influencing factors for PGF and prolonged thrombocytopenia are numerous and vary over time after allo‐HCT, model‐based platelet reconstitution predictions could be a valuable method to support clinical hematologists in the identification of patients with post‐HCT thrombocytopenia. The development of highly predictive models, however, requires longitudinal clinical data from a large cohort of patients undergoing allo‐HCT. The XplOit project established novel semantic data integration tools and a supporting IT‐infrastructure to facilitate the data protection‐compliant harmonization and usage of real‐world data from heterogenous sources for the development, validation, and application of mathematical models predicting individual outcomes following allo‐HCT.^15^, ^16^, ^17^

This study within the XplOit consortium developed a model of long‐term platelet reconstitution (PLT‐R) in adult patients undergoing allo‐HCT that allows the prediction of individual platelet counts for up to 180 days after allo‐HCT from day +28. To translate the model for use in clinical practice, it was implemented into a publicly accessible web‐based demonstrator.

## MATERIALS AND METHODS

### Patients

We analyzed data obtained during clinical care from adult patients who underwent their first allo‐HCT at the Department of Hematology and Stem Cell Transplantation of the University Hospital of Essen. Data were collected from a large retrospective cohort of 1710 patients with allo‐HCT between January 2005 and August 2017 and from 407 patients included in the prospective non‐interventional XplOit study with allo‐HCT between September 2017 and June 2020. The XplOit study was conducted following German legislation and the revised Helsinki Declaration. Study performance and data acquisition were evaluated by the institutional review boards of the University Duisburg‐Essen (Protocol No. 17‐7576‐BO) and the medical association of the Saarland (Protocol No. 33/17). Written consent was obtained from all patients included in the prospective non‐interventional part of the XplOit study, which was registered in the German Clinical Trials Register (DRKS), registration No. DRKS00026643. A detailed description of dataset preparation is provided in the **Supplementary Materials and Methods**.

### Model development

For the development of our model, we leveraged an established semi‐mechanistic model of chemotherapy‐induced myelosuppression^18^ and additionally incorporated the transplantation of donor‐derived hematopoietic stem cells (HSCs). The drug effect of the conditioning regimen was incorporated for the individual time between the first and last dosing of any chemotherapeutic drug or radiotherapy. For this, we tested constant and time‐dependent drug effects, as well as kinetic‐pharmacodynamic models. In the absence of platelet transfusion records, we explored values from 10 × 10^9^/L to 15 × 10^9^/L for the imputation of platelet transfusions between day +1 and day +30 post‐HCT, with a threshold of 12 × 10^9^/L optimally improving the model fit. This threshold aligns with the University Hospital Essen's guideline, which typically initiates transfusions for platelet counts below 10 × 10^9^/L, while it acknowledges that some clinical scenarios, such as severe infections or heightened bleeding risks, might require higher thresholds.

All available platelet measurements between day −30 and day +180 post‐HCT were included for modeling. A non‐linear‐mixed‐effects model implemented in NONMEM® (version 7.4.3, ICON Development Solutions, Ellicott City, MD, USA) was used for population analysis, simulation, and prediction. In addition, model selection was based on statistical criteria, precision of parameter estimates, goodness‐of‐fit plots, and predictive performance assessments. Data preparation, statistical analyses, and graphical presentation were carried out using R (version 3.6.3, R Foundation for Statistical Computing, Vienna, Austria). For further details on model development and versions of used R packages, see the **Supplementary Materials and Methods**.

### Covariate analysis

For the pre‐selection of covariates to be tested, we graphically examined the relationships of individual model parameters and continuous and categorical variables using scatter and box‐and‐whisker plots, respectively. For each combination of variable and model parameter that presented a pattern or trend in these plots, we subsequently tested the effect of the respective covariate on the corresponding model parameter. **Figures** **S1–S8** display the exploratory plots for relevant model parameters. **Table** **S1** lists the static variables that were graphically examined including the percentages of missing values. A list of all model parameter‐covariate combinations that were finally tested is provided in **Table** **S2**. **Table** **S3** lists the laboratory markers that were tested as time‐dependent covariates including the percentages of patients without any measurement of the respective laboratory marker. Further details on covariate analysis can be found in the **Supplementary Materials and Methods**.

### Prediction of individual platelet counts after allo‐HCT

The final model was used to predict platelet–time profiles for patients in the test (retrospective data, *n* = 518) and validation (prospective data, *n* = 383) dataset during internal and external model validation. Here, we evaluated the prediction of future platelet counts from days +7, + 14, +21, and +28 post‐HCT. Using Bayesian forecasting and the model parameters derived from the training dataset (retrospective data, *n* = 1,048) as priors, platelet–time profiles were predicted on the basis of early phase data. Individual model parameters were obtained using Maximum a posteriori (MAP) estimation and patient data that were collected pre‐HCT (i.e., patient and transplantation characteristics, platelet counts), as well as, depending on the prediction day, weekly post‐HCT platelet counts, corresponding to days +7, +14, +21, and +28. Information on platelet transfusions were included until the prediction day. Subsequently, the individual model parameters were used to generate platelet–time profiles for 180 days post‐HCT. We investigated the predictive performance using graphical and quantitative criteria. Furthermore, we assessed the model's ability to predict grade 2 thrombocytopenia (platelet count < 75 × 10^9^/L),^19^ with the mean value of the last 12 platelet measurements during the prediction period being used to determine thrombocytopenia. The individual prediction period was defined by day +29 and the latest platelet measurement within the first 180 days post‐HCT.

During the model evaluation, we performed a fivefold cross‐validation to examine the potential influence of splitting the retrospective cohort in training and test datasets on the estimation of model parameters. Details on the cross‐validation procedure and predictive performance assessment are provided in the **Supplementary Materials and Methods**.

### Web‐based demonstrator of the platelet reconstitution model

To provide access to the PLT‐R model and personalized predictions of post‐HCT platelet counts for informational purposes, the final model was implemented into a web‐based demonstrator using R (version 4.3.0) and the R package shiny (version 1.7.5.1).

### Data availability

The data used in this study involve sensitive personal health information. Due to the high dimensionality and the inclusion of longitudinal data, it cannot be fully anonymized and published without the risk of re‐identification. Any requests for access to the data may be submitted to the University Hospital of Essen and are subject to approval by the data protection officer and ethics committee. To enable the independent reproduction of our study, however, details on dataset preparation and the NONMEM® control file with all model equations are included in the **Supplementary Materials and Methods**.

## RESULTS

### Patient characteristics

**Figure 1:**
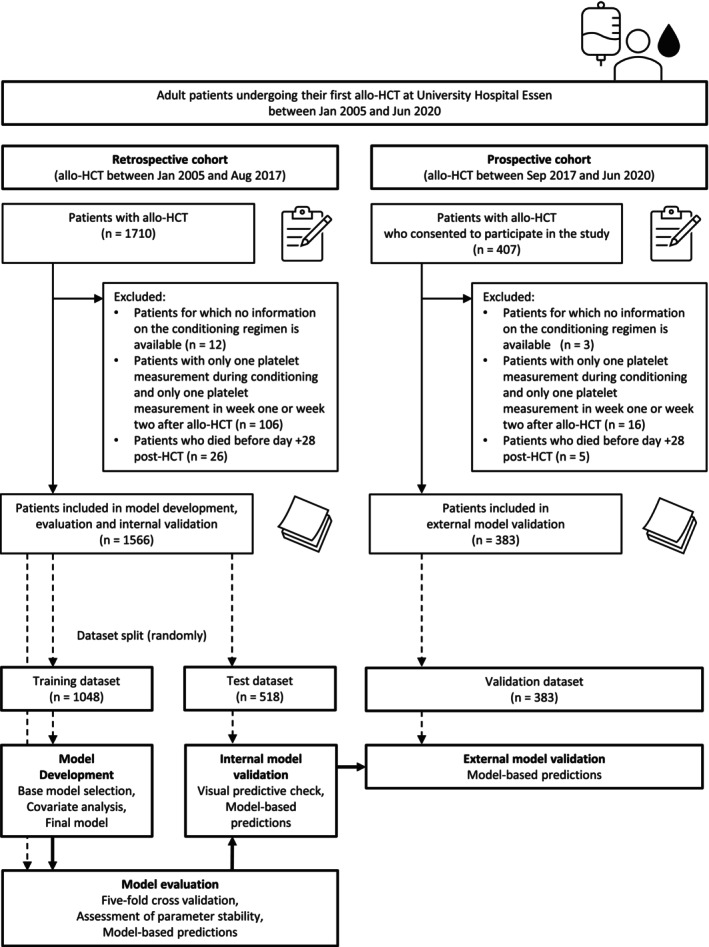
Illustration of the patient selection process (upper panel) and study flowchart (lower panel).

The final dataset included 1949 patients, with the retrospective cohort randomly split up in a 2:1 ratio into a training (*n* = 1,048) and test (*n* = 518) dataset, and the prospective cohort used as validation (*n* = 383) dataset. The retrospective and prospective cohorts presented a median of 58 and 56 (range 26–187 and 24–182) platelet measurements during the first 180 days after allo‐HCT, respectively. **Figure** **1** shows the patient selection process and study flowchart.

**Figure 2:**
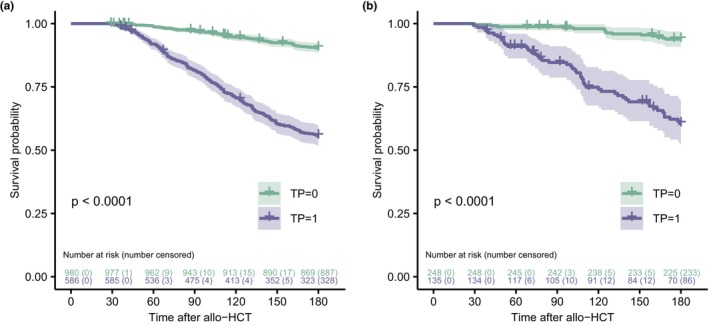
Kaplan–Meier curves and at‐risk tables for overall survival in patients without and with thrombocytopenia (TP) from the retrospective (**a**) and prospective (**b**) cohort. Patients who were alive at day +180 or the time of latest platelet measurement during the first 180 days after allo‐HCT were censored. *P*‐values were derived from stratified log‐rank tests. TP, mean platelet count < 75 × 10^9^/L during the 12 latest platelet measurements within 180 days after allo‐HCT.

**Table** **1** provides a comparison of baseline patient characteristics. A post hoc Dunn test indicated statistically significant differences (*P* < 0.05) between the retrospective and prospective cohort for patient age (median 54 vs. 57 years) and donor age (median 36 vs. 31 years). Furthermore, the prospective cohort was characterized by a higher percentage of sibling transplants, identical HLA‐matches, and peripheral blood stem cells. Tacrolimus was more often used for baseline immune suppression in the prospective cohort. Importantly, the incidence of grade 2 thrombocytopenia was evenly distributed between the cohorts and occurred in 37% of all patients. Overall survival rates of patients with grade 2 thrombocytopenia were significantly lower (*P* < 0.0001) in each cohort, as indicated by Kaplan–Meier curves and stratified log‐rank tests (**Figure** **2**). For the survival analyses, we censored patients who were alive at day +180 or at the time of the latest platelet measurement during the first 180 days after allo‐HCT.

**Table 1:** Baseline patient characteristics

Variable	Retrospective data		Prospective data		
	Training dataset	Test dataset	Validation dataset	*P*‐value^a^	Complete dataset
	(*n* = 1,048)	(*n* = 518)	(*n* = 383)		(*n* = 1949)
	Median (range)	Median (range)	Median (range)		Median (range)
Patient age (years)	54 (17, 76)	54 (17, 74)	57 (18, 75)	< 0.001	54 (17, 76)
Donor age (years)	36 (12, 80)	36 (13, 73)	31 (16, 70)	< 0.001	35 (12, 80)
	***n* (%)**	***n* (%)**	***n* (%)**		***n* (%)**
Patient sex					
Male	588 (56.11)	304 (58.69)	220 (57.44)	0.615	1,112 (57.05)
Female	460 (43.89)	214 (41.31)	163 (42.56)		837 (42.95)
Sex matching					
Matched	682 (65.08)	323 (62.36)	251 (65.54)	0.425	1,256 (64.44)
Female to male	140 (13.36)	78 (15.06)	61 (15.93)		279 (14.32)
Male to female	226 (21.56)	117 (22.59)	71 (18.54)		414 (21.24)
Donor relationship					
Related	280 (26.72)	121 (23.36)	122 (31.85)	0.017	523 (26.83)
Unrelated	768 (73.28)	397 (76.64)	261 (68.15)		1,426 (73.17)
HLA matching^b^					
Identical (10/10)	801 (76.43)	389 (75.1)	318 (83.03)	0.011	1,508 (77.37)
Not identical	247 (23.57)	129 (24.9)	65 (16.97)		441 (22.63)
CMV serostatus patient					
Negative	452 (43.13)	201 (38.8)	158 (41.25)	0.114	811 (41.61)
Positive	579 (55.25)	304 (58.69)	218 (56.92)		1,101 (56.49)
Unknown	5 (0.48)	7 (1.35)	1 (0.26)		13 (0.67)
Stem cell source					
PBSC	956 (91.22)	484 (93.44)	375 (97.91)	< 0.001	1815 (93.12)
Bone marrow	84 (8.02)	33 (6.37)	8 (2.09)		125 (6.41)
PBSC and Bone marrow	7 (0.67)	1 (0.19)			8 (0.41)
Diagnosis					
Acute myeloid leukemia	476 (45.42)	240 (46.33)	191 (49.87)	0.399	907 (46.54)
Acute lymphoblastic leukemia	106 (10.11)	60 (11.58)	36 (9.4)		202 (10.36)
Non‐Hodgkin lymphoma	106 (10.11)	50 (9.65)	27 (7.05)		183 (9.39)
Myelodysplastic syndromes	97 (9.26)	48 (9.27)	46 (12.01)		191 (9.8)
Myeloproliferative Disorder	72 (6.87)	36 (6.95)	26 (6.79)		134 (6.88)
Chronic myeloid leukemia	59 (5.63)	18 (3.47)	14 (3.66)		91 (4.67)
Others^c^	132 (12.6)	66 (12.74)	43 (11.23)		241 (12.37)
Disease stage^d^					
Early	330 (31.49)	156 (30.12)	114 (29.77)	0.475	600 (30.79)
Advanced	576 (54.96)	296 (57.14)	179 (46.74)		1,051 (53.93)
Unknown	142 (13.55)	66 (12.74)	90 (23.5)		298 (15.29)
Conditioning treatment					
Myeloablative conditioning	454 (43.32)	211 (40.73)	160 (41.78)	0.331	825 (42.33)
Reduced intensity conditioning	557 (53.15)	284 (54.83)	215 (56.14)		1,056 (54.18)
Other	37 (3.53)	23 (4.44)	8 (2.09)		68 (3.49)
ATG treatment					
Yes	589 (56.2)	299 (57.72)	231 (60.31)	0.374	1,119 (57.41)
Daily dose (mg)	800 (350, 2,700)	800 (440, 3,700)	800 (430, 2,500)	0.066	800 (350, 3,700)
Baseline immunosuppression					
Cyclosporine A	988 (94.27)	482 (93.05)	261 (68.15)	< 0.001	1731 (88.81)
Tacrolimus	2 (0.19)	5 (0.97)	108 (28.2)		115 (5.9)
Triple suppression	27 (2.58)	19 (3.67)	6 (1.57)		52 (2.67)
Graft manipulation	17 (1.62)	6 (1.16)	2 (0.52)		25 (1.28)
Other	14 (1.34)	6 (1.16)	6 (1.57)		26 (1.33)
Acute GvHD					
Grades 0–1	638 (60.88)	281 (54.25)	250 (65.27)	0.003	1,169 (59.98)
Grades 2–4	410 (39.12)	237 (45.75)	133 (34.73)		780 (40.02)
Thrombocytopenia (TP)^e^	394 (36.35)	192 (37.07)	135 (35.25)	0.881	721 (36.99)

ATG, anti‐thymocyte globulin; CMV, cytomegalovirus; GvHD, graft‐versus‐host disease; HLA, human leukocyte antigen; PBSC, peripheral blood stem cell.

^a^ The continuous variables were compared using the Kruskal‐Wallis test, and differences in frequency between groups were compared using the chi‐square test.

^b^ Human leukocyte antigen (HLA)‐A, ‐B, ‐C, ‐DRB1, ‐DQB1 were considered.

^c^ Other diagnoses: Multiple Myeloma, chronic lymphocytic leukemia, non‐malignant hematological diseases, chronic myelomonocytic leukemia, Hodgkin lymphoma, other hematologic malignancies, congenital hematologic disorder, non‐hematologic malignant disorder, hemoglobinopathy.

^d^ Early stages: De‐novo acute myeloid leukemia in 1st remission, acute lymphoblastic leukemia in 1st remission, myelodysplastic syndromes with single lineage dysplasia, and myelodysplastic syndromes with single lineage dysplasia and ring sideroblasts, chronic myeloid leukemia in 1st chronic phase. Advanced stages: All other stages that did not correspond to early stages, such as acute myeloid leukemia in 2nd remission.

^e^ Thrombocytopenia (TP): mean platelet count < 75 × 10^9^/L, derived from the last 12 platelet measurements within 180 days after allo‐HCT.

### The platelet reconstitution model

**Figure 3:**
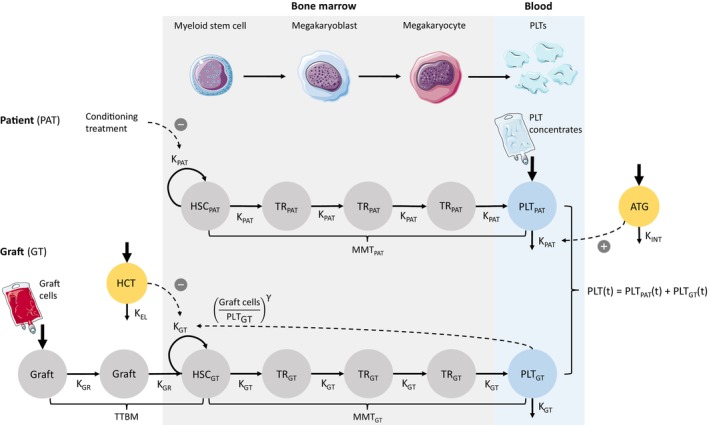
Schematic representation of the PLT‐R model. The model describes post‐HCT platelet counts as the sum of predicted blood cells derived from hematopoietic stem cells (HSCs) of both the patient (PAT) and the graft (GT). It includes 14 compartments: proliferating HSCs, three differentiation transit compartments (TR), and circulating platelets (PLT) for both PAT and GT; a virtual ATG concentration (ATG), two compartments for transplanted HSCs (Graft), and an effect compartment (HCT). HSCs progress through differentiation stages in the bone marrow (light gray) before entering the peripheral blood (light blue). Key parameters for GT‐derived platelet kinetics include the baseline platelet count post‐HCT (Graft cells), the HCT effect, the mean maturation time (MMT), and the feedback parameter gamma. The model captures the inhibitory effect of the conditioning and a feedback loop from PLT_GT_ to proliferating HSC_GT_. Dummy inputs simulate fluctuations in PLT_PAT_ due to apheresis cells (day +1) and platelet concentrates (first 30 days post‐HCT). ATG, anti‐thymocyte globulin; GT, graft; HSC, hematopoietic stem cell; PAT, patient.

The final model reflects pre‐existing knowledge of the biological processes of thrombopoiesis (**Figure** **3**, upper panel), modified from an established myelosuppression model, in which proliferating HSCs move through three differentiation stages in the bone marrow before entering the peripheral blood, where they are cleared.^18^ Considering the following assumptions for the allo‐HCT setting, which are (1) the pre‐HCT conditioning treatment depletes the HSCs of the patient, and (2) the differentiation of transplanted HSCs facilitates the post‐HCT platelet reconstitution, the presented model incorporates two submodels, respectively representing the differentiation of HSCs from the patient (**Figure** **3**, middle panel) and the graft (**Figure** **3**, lower panel). Subsequently, platelet counts over time (PLT(*t*)) were modeled (**Eq**. **1**) as the sum of platelets from the patient (PAT) and the graft (GT).

(1) $$\mathrm{PLT}\left(t\right)={\mathrm{PLT}}_{\mathrm{PAT}}\left(t\right)+{\mathrm{PLT}}_{\mathrm{GT}}\left(t\right)$$

This structure allowed the characterization of pre‐HCT platelet kinetics within the patient model and post‐HCT platelet kinetics within the graft model. Certain model parameters, such as baseline platelet counts and mean maturation times (MMTs), are relevant to both pre‐ and post‐HCT platelet kinetics but may exhibit individual variation across these periods; hence, these were estimated separately for each submodel. The individually estimated baseline platelet count pre‐HCT (HSC) was used to initialize the compartment of proliferating HSCs, the three transit compartments (TR1–3), and the compartment of blood cells (PLT) within the patient model. In both submodels, the transit rates (*K*) were defined as $\left(n+1\right)/\mathrm{MMT}$, whereas *n* corresponds to the number of transit compartments, which was assumed to be 3 for both the patient and the graft model. The final model parameters, optimized using the training dataset, were estimated precisely and physiologically plausible (**Table** **S4**).

*Patient model*: The following system of ordinary differential equations (ODEs) defines the patient model (**Eqs**. **(2)**, **(3)**, **(4)**, **(5)**, **(6)**, **(7)**):

(2) $$\begin{array}{c} \frac{{\text{dHSC}}_{\mathrm{PAT}}}{dt}=K_{\mathrm{PAT}}\times \left(1-\text{DRUG}\left(t\right)\right)\times {\mathrm{HSC}}_{\mathrm{PAT}}\left(t\right)\\ \;-K_{\mathrm{PAT}}\times {\mathrm{HSC}}_{\mathrm{PAT}}\left(t\right) \end{array}$$

(3) $$\frac{\mathrm{dTR}1_{\mathrm{PAT}}}{dt}=K_{\mathrm{PAT}}\times {\mathrm{HSC}}_{\mathrm{PAT}}\left(t\right)-K_{\mathrm{PAT}}\times \mathrm{TR}1_{\mathrm{PAT}}\left(t\right)$$

(4) $$\frac{\mathrm{dTR}2_{\mathrm{PAT}}}{dt}=K_{\mathrm{PAT}}\times \mathrm{TR}1_{\mathrm{PAT}}\left(t\right)-K_{\mathrm{PAT}}\times \mathrm{TR}2_{\mathrm{PAT}}\left(t\right)$$

(5) $$\frac{\mathrm{dTR}3_{\mathrm{PAT}}}{dt}=K_{\mathrm{PAT}}\times \mathrm{TR}2_{\mathrm{PAT}}\;\left(t\right)-K_{\mathrm{PAT}}\times \mathrm{TR}3_{\mathrm{PAT}}\left(t\right)$$

(6) $$\begin{array}{c} \frac{{\text{dPLT}}_{\mathrm{PAT}}}{dt}=K_{\mathrm{PAT}}\times \mathrm{TR}3_{\mathrm{PAT}}\left(t\right)-K_{\mathrm{PAT}}\times {\mathrm{PLT}}_{\mathrm{PAT}}\left(t\right)\\ \;\times \left(1+\alpha \times \mathrm{ATG}\left(t\right)\right) \end{array}$$

(7) $$\frac{\text{dATG}}{dt}=-K_{\mathrm{INT}}\times \mathrm{ATG}\left(t\right)$$

The inhibitory effect of the conditioning (DRUG(*t*)) was incorporated during the conditioning treatment, as depicted in **Eq**. **8**:

(8) DRUGt=ρ,t≥tstart0,t>tstop

with *t*_start_ and *t*_stop_ indicating the start and end time of conditioning.

The baseline platelet count was estimated at 90.5 × 10^9^/L with large inter‐patient variability of 115.0% coefficient of variation (%CV). The MMT was estimated at 6.1 days. During the conditioning treatment, the HSC proliferation rate $K_{\mathrm{PAT}}$ was decreased by 94.5% (typical value of 0.945 for *ρ*; 152.3 %CV), which led to a delayed decrease in platelet counts. Preliminary data analysis showed two distinct patterns of platelet kinetics in response to conditioning. Patients receiving anti‐thymocyte globulin (ATG) exhibited an excessive decrease in platelet counts immediately after ATG treatment, whereas patients without ATG showed a less pronounced decrease in platelet counts (**Figure** **S9**). We established a kinetic‐pharmacodynamic model^20^ to incorporate the ATG‐effect and implemented ATG kinetics based on the individual dosing regimen (**Eq**. **7**). Assuming first‐order elimination (*K*_INT_), the individually predicted ATG concentrations (ATG(*t*)) were linked to platelet counts (PLT_PAT_(*t*)) via a linear concentration–effect model with parameter α, resulting in increased platelet eliminations during ATG treatment (**Eq**. **6**). Platelet counts increased on day +1, likely due to donor‐derived platelets transmitted with the stem cell graft. To account for this phenomenon, cells were added to the platelet compartment on day 0, increasing platelet counts on day +1 by 20.1 × 10^9^/L (289.7 %CV). Likewise, cells were added to the platelet compartment at each administration of platelet transfusions, increasing platelet counts on the following day by 10.6 × 10^9^/L (67.9 %CV).

*Graft model*: The following system of ODEs defines the graft model (**Eqs**. **(9)**, **(10)**, **(11)**, **(12)**, **(13)**, **(14)**, **(15)**, **(16)**):

(9) $$\frac{\text{dHCT}}{dt}=-K_{\mathrm{EL}}\times \mathrm{HCT}\;\left(t\right)$$

(10) $$\frac{\text{dGraft}1}{dt}=-K_{\mathrm{GR}}\times \text{Graft}1\;\left(t\right)$$

(11) $$\frac{\text{dGraft}2}{dt}=K_{\mathrm{GR}}\times \text{Graft}1\left(t\right)-K_{\mathrm{GR}}\times \text{Graft}2\left(t\right)$$

(12) $$\begin{array}{c} \frac{{\text{dHSC}}_{\mathrm{GT}}}{dt}=K_{\mathrm{GT}}\times {\left(\frac{\text{Graft}1_{0}}{{\mathrm{PLT}}_{\mathrm{GT}}\left(t\right)}\right)}^{\gamma }\times \left(1-\mathrm{HCT}\left(t\right)\right)\times {\mathrm{HSC}}_{\mathrm{GT}}\;\left(t\right)\\ \;-K_{\mathrm{GT}}\times {\mathrm{HSC}}_{\mathrm{GT}}\left(t\right)+K_{\mathrm{GR}}\times \text{Graft}2\left(t\right) \end{array}$$

(13) $$\frac{\mathrm{dTR}1_{\mathrm{GT}}}{dt}=K_{\mathrm{GT}}\times {\mathrm{HSC}}_{\mathrm{GT}}\left(t\right)-K_{\mathrm{GT}}\times \mathrm{TR}1_{\mathrm{GT}}\left(t\right)$$

(14) $$\frac{\mathrm{dTR}2_{\mathrm{GT}}}{dt}=K_{\mathrm{GT}}\times \mathrm{TR}1_{\mathrm{GT}}\left(t\right)-K_{\mathrm{GT}}\times \mathrm{TR}2_{\mathrm{GT}}\left(t\right)$$

(15) $$\frac{\mathrm{dTR}3_{\mathrm{GT}}}{dt}=K_{\mathrm{GT}}\times \mathrm{TR}2_{\mathrm{GT}}\left(t\right)-K_{\mathrm{GT}}\times \mathrm{TR}3_{\mathrm{GT}}\left(t\right)$$

(16) $$\frac{{\text{dPLT}}_{\mathrm{GT}}}{dt}=K_{\mathrm{GT}}\times \mathrm{TR}3_{\mathrm{GT}}\left(t\right)-K_{\mathrm{GT}}\times {\mathrm{PLT}}_{\mathrm{GT}}\left(t\right)$$

Graft cells ($\text{Graft}1_{0}$) were added to a graft compartment (Graft1) on day 0, reflecting the transplantation of donor‐derived HSCs. The mean time for transition from Graft1 to the HSC compartment (time to bone marrow, TTBM) was estimated at 1.5 days. Here, the transit rate (*K*_GR_) was defined as 2/TTBM. Representing the baseline platelet count post‐HCT ($\text{Graft}1_{0}$), the number of graft cells was estimated at 72.4 × 10^9^/L with moderate inter‐patient variability of 82.9 %CV. The MMT post‐HCT was estimated at 7.0 days (90.9 %CV). The feedback exponent γ was estimated at 0.19 (48.7 %CV) for the ratio of graft cells and predicted platelets, introducing feedback from blood cells (PLT_GT_(*t*)) to proliferating HSCs (HSC_GT_(*t*)) and accounting for the overshoot of platelets after the platelet nadir. This feedback represents the endogenous regulation of cell proliferation, for example, by colony‐stimulating factors.^18^ Low cell counts during the platelet nadir increased this feedback, resulting in overpredicted platelet counts. Additionally, the duration of the platelet nadir phase was highly variable between patients (**Figure** **S9**), likely caused by an effect of the transplantation. This HCT‐effect was well described by an indirect‐response model linking the individually predicted HCT‐effect to the proliferation of donor‐derived HSCs. We introduced the HCT‐effect compartment with zero‐order input on day 0 (**Eq**. **9**). The HCT‐effect was assumed to decline over time and a first‐order elimination rate (*K*_EL_) was estimated at 0.353 days^−1^, corresponding to an HCT‐effect elimination half‐life of 2.0 days. The mean HCT‐effect on day 0 was estimated at 4.22 (87.1 %CV).

The accurate descriptive performance of the final model is demonstrated in overall goodness‐of‐fit plots (**Figure** **S10**) and individual profiles (**Figure** **S11**). **Figure** **S12** shows the simulated platelet–time profile for a typical patient, delineating HSCs and blood cells originating from the patient and the graft, illustrating that the drug effect depletes the patient‐derived HSCs, leading to a decrease of patient‐derived blood cells. After day 0, HSCs from the graft start proliferation, followed by the increase of blood cells originating from the graft. Further diagnostic plots are provided in **Figure** **S10**.

### Effects of covariates on platelet counts after allo‐HCT

**Figure 4:**
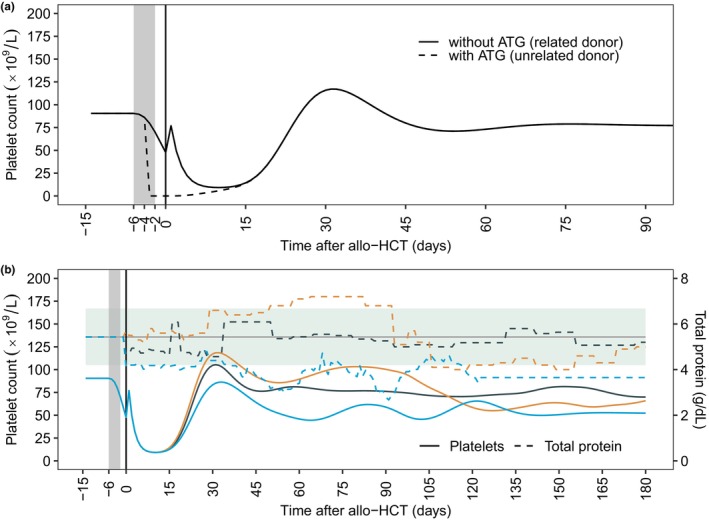
Effects of covariates on platelet counts after allo‐HCT. (**a**) Simulated platelet–time profiles for typical patients without and with a daily dose of 800 mg ATG from day −4 to day −2, respectively representing patients with allo‐HCT from related and unrelated grafts. (**b**) Simulated platelet–time profiles from observed total protein levels of three randomly selected patients from the test dataset. The gray horizontal line and the green background represent the median and 10th and 90th percentile total protein values. (**a, b**) The gray background represents the period of the conditioning from day −6 to day −2. The black vertical line represents the transplantation day. The small peaks immediately after stem cell transplant refer to the increase in platelet counts on day +1, with donor‐derived platelets transmitted with the stem cell graft possibly contributing to this phenomenon. (**b**) Patients represent typical patients without ATG treatment. ATG, anti‐thymocyte globulin.

The effects of significant covariates for post‐HCT platelet reconstitution were simulated for typical patients with conditioning between day −6 and day −2 (**Figure** **4**). As previously mentioned, ATG influenced pre‐HCT platelet kinetics and was incorporated early in the model development. The use of 800 mg ATG from day −4 to day −2 induced an immediate decrease of platelet counts, leading to an earlier platelet nadir in comparison to the absence of ATG (day −3 vs. day +5; **Figure** **4** **a**). Donor relation significantly influenced the increase in platelet counts on day +1. In patients with sibling HCT, the increase in platelet counts on day +1 was 2.75 times higher compared with patients with unrelated donors (**Figure** **4** **a**). The extensive exploration of further covariates, such as diagnosis (myeloproliferative disorders vs. others), donor type (matched‐related or mismatched‐related vs. others), HLA matching (identical vs. others), stem cell source (bone marrow vs. others), or CD34+ stem cell dose (e.g., < 5 × 10^6^ cells vs. others), among other variables (**Table** **S2**), revealed minimal impact of patient and transplantation characteristics on post‐HCT platelet counts, except for total protein.

Total protein demonstrated continuous effects on the proliferation of HSCs in the graft model. To describe this, the individual and time‐dependent total protein level was centered around the median (5.43 g/dL) and the model exponent was estimated at 0.197. We simulated platelet–time profiles of typical patients without ATG using the observed total protein levels of three randomly selected patients from the test dataset, demonstrating platelet depletion or growth with decreasing or increasing total protein levels (**Figure** **4** **b**).

### Prediction of individual platelet counts after allo‐HCT

The available patient data before allo‐HCT and on days +7, +14, +21, and +28 post‐HCT was used to estimate individual model parameters and predict platelet count–time profiles for the period in which platelet data were available and a maximum of 180 days after allo‐HCT. Each prediction scenario included information on ATG and donor relation. Total protein levels were included on days +7, +14, +21, and +28 post‐HCT, whereas the total protein effect remained uninformed censored (= no effect) after the respective prediction day.

**Figure 5:**
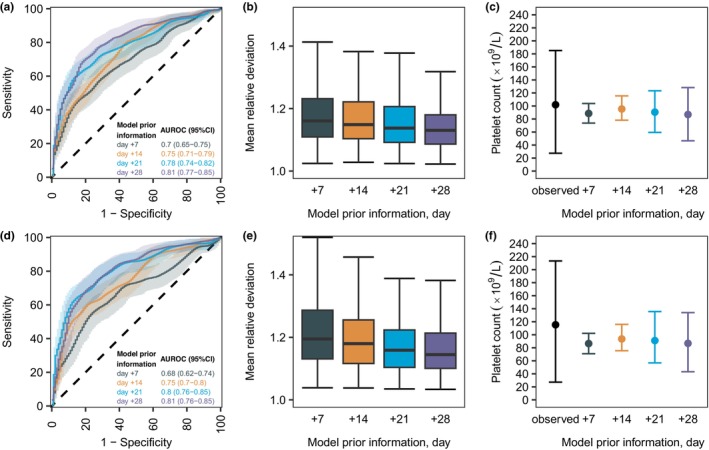
Predictive performance of the PLT‐R model for internal (upper panel) and external (lower panel) model validation. (**a, d**) ROC curves and respective AUROC values are shown. The shaded areas show the 95% confidence interval for AUROC values calculated from bootstrap analysis (*n* = 1,000). (**b, e**) Box‐plots of mean relative deviations calculated from individual predicted platelet–time profiles. (**c, f**) Mean, 10th and 90th percentile values of all mean observed, and all mean individual predicted platelet counts during the individual prediction period are shown. (**a–f**) Colors show different levels of model prior information. AUROC, area under the receiver‐operating characteristic curve; ROC, receiver‐operating characteristic.

**Figure 6:**
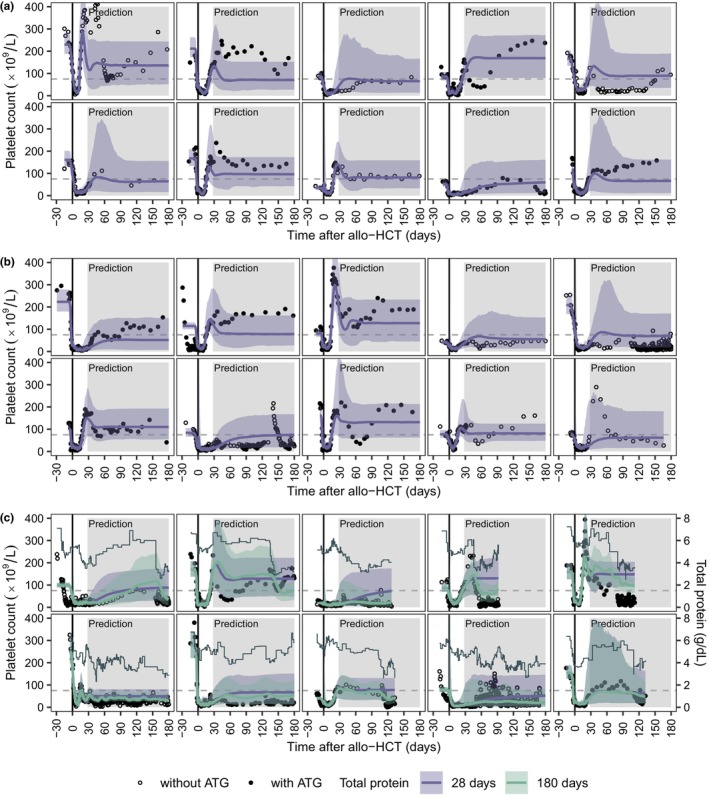
Individual prediction of platelet–time profiles from day + 28 after allo‐HCT. (**a, b**) Platelet–time profiles of 10 randomly selected patients from the test (**a**) and validation (**b**) dataset with a minimum observation period of 80 days post‐HCT. (**c**) Platelet–time profiles of 10 randomly selected patients from the test dataset with at least 12 total protein measurements below the 10th percentile value of 4.2 g/dL and a minimum observation period of 80 days post‐HCT. (**a–c**) Black circles and dots present observed platelet counts of patients without and with ATG treatment, respectively. The lines and shaded areas display the individual predicted platelet counts and the 95% prediction intervals. Total protein information for 28 and 180 days after allo‐HCT is depicted in purple and green, respectively. Gray solid lines indicate measured total protein levels, while dashed gray lines represent the platelet count of 75 × 10^9^/L. ATG, anti‐thymocyte globulin.

On day +28, the model reached AUROC values of 0.81 (95% confidence interval (CI): 0.77–0.85) and 0.81 (95%CI: 0.76–0.85) for the prediction of grade 2 thrombocytopenia during internal and external validations, respectively (**Figure** **5** **a,d**). Model‐based predictions from earlier timepoints presented reduced predictive performance (**Figure** **5** **a,d**). However, only the predictions from day +7 resulted in AUROC values ≤ 0.7. Overall, mean relative deviations were adequate (< 2, **Figure** **5** **b,e**), and mean individual predictions were in good agreement with mean observations (**Figure** **5** **c,f**). On day +28, the model predicted the individual long‐term platelet reconstitution after allo‐HCT adequately, as can be seen for 10 randomly selected patients from the test and validation dataset (**Figure** **6** **a,b**). However, on day +28, the model was unable to accurately capture fluctuations in individual platelet counts during the prediction period. To investigate this issue, the prognostic importance of total protein was graphically examined. Platelet–time profiles were predicted from day +28, and additional total protein information was included throughout the prediction period. As illustrated for 10 randomly selected patients from the test dataset, continuous total protein information could estimate notable changes in platelet counts for the time after day +28 post‐HCT (**Figure** **6** **c**).

The cross‐validation results demonstrated consistency in final parameter estimates across all five folds (**Table** **S5**). Performance metrics for day +28 were comparable between internal model validation and cross‐validation (AUROC: 0.81 versus 0.78–0.84), as well as between the folds (**Figure** **S13**).

### Web‐based demonstrator of the platelet reconstitution model

A user‐friendly demonstrator of the final model was set up at https://hsct.precisiondosing.de. The integrated example data allows the prediction of post‐HCT platelet counts for three characteristic patients. Additionally, the upload and use of external data is supported, which involves the following data: patient‐ and transplantation characteristics (patient weight, donor relation, allo‐HCT date, conditioning period, and if used, ATG dose and treatment period), platelet counts (before allo‐HCT, and at least on day +7), total protein measurements (recommended, but not essential), and platelet transfusion dates. Data input and prediction output are exemplified in **Figures** **S14** **and** **S15**.

## DISCUSSION

Contributing to the existing knowledge, this study reports significantly reduced (*P* < 0.0001) overall survival rates for patients with grade 2 thrombocytopenia after allo‐HCT. To support clinical decision‐making, this study developed and validated a model for long‐term PLT‐R after allo‐HCT that allows individual predictions of platelet counts for up to 180 days from early post‐HCT timepoints. For clinical implementation, a demonstrator of the model was established at https://hsct.precisiondosing.de. Supporting model‐based predictions from external data, the demonstrator could translate model insights into interpretable results for health care professionals. While the model is not assigning post‐HCT thrombocytopenia to a specific cause, such as acute GvHD or viral reactivation, it could serve as a preliminary screening tool. Patients who were identified as having insufficient platelet counts could undergo frequent monitoring and extensive diagnostics to determine the underlying causes, which, if treatable, could mitigate the risk of post‐HCT complications. Most importantly, model‐based predictions might be helpful to identify patients at risk of prolonged thrombocytopenia or PGF, and thus, assist the early decision for thrombopoietin receptor agonists^21^, ^22^ or the timely initiation of donor stem cell boosts.^23^, ^24^

The developed model achieved adequate predictive performance of grade 2 thrombocytopenia from day +7, with increasing accuracy when providing additional measurements on days +14, +21, and +28. To further improve the predictions, it is possible to include not only the clinical data collected on specific timepoints post‐HCT, but also all available platelet measurements between day 0 and the prediction day. We were able to investigate the model performance using prospective data from a contemporary patient cohort during external model validation, which demonstrated similar performance metrics when compared with internal model validation. Whereas post hoc tests revealed significant differences between retrospective and prospective cohorts in terms of HLA‐match and donor relationship, factors previously linked to delayed platelet reconstitution after allo‐HCT,^4^, ^12^ the good predictive performance during external validation indicates model robustness. Nevertheless, recognizing the dynamic field of stem cell transplantation and the emergence of new treatment options, such as post‐transplant cyclophosphamide, we acknowledge the importance of updating our model to reflect these advancements. A future perspective might involve routinely adjusting the model with new data and assess its predictive performance using the initial and updated priors.

Simulations of the final model indicated an average time to platelet engraftment — defined as the first of three consecutive days with platelet counts ≥ 20 × 10^9^/L — of 17 days, which is consistent with studies reporting platelet engraftment from day +12 to day +18 in patients with allo‐HCT between 2005 and 2017.^11^, ^25^, ^26^, ^27^ The HCT‐effect, feedback exponent γ and MMT post‐HCT determine the individual time to platelet engraftment in the presented model. Conditioning may contribute to the HCT‐effect through a drug‐induced tissue damage, resulting in elevated levels of inflammatory mediators in the bone marrow, which may persist beyond the conditioning period. For instance, TNF‐α, IFN‐γ, and reactive oxygen species have been reported to negatively impact HSCs.^28^, ^29^, ^30^, ^31^ The average duration of the HCT‐effect (9.8 days) aligns with previously reported low levels of immature platelet fractions for 11 days post‐HCT.^32^ Furthermore, the average γ was comparable to results from modeling analyses of platelets in patients without allo‐HCT,^33^, ^34^, ^35^, ^36^, ^37^, ^38^ and the average MMT post‐HCT (7 days) is comparable to the reported platelet lifespan of 8–9 days in healthy individuals.^39^

While previous studies focused on single timepoints for the evaluation of risk factors for post‐HCT thrombocytopenia,^4^, ^5^, ^12^ our model integrates longitudinal platelet data and allows the investigation of both static and dynamic variables. Importantly, this study clarified the impact of ATG GvHD prophylaxis on post‐allo‐HCT platelet reconstitution. ATG rapidly decreased platelet counts in a linear concentration–effect relationship and the increase in platelet counts on day +1 was higher in patients receiving sibling HCT, who typically did not receive ATG. These findings indicate that ATG binds to platelets, in agreement with previous studies.^40^ However, while the secondary failure of PLT‐R was previously linked to *in vivo* T‐cell depletion by ATG or alemtuzumab,^8^ the covariate analysis on our data revealed no significant long‐term effects of ATG.

Total serum protein had continuous effects on the proliferation of donor‐derived HSCs, potentially explained via thrombopoietin synthesis in the liver, which is involved in the endogenous regulation of platelets.^41^ Albumin, constituting the majority of total blood proteins, accounted for a smaller percentage of the inter‐patient variability on graft cells and led to a poorer predictive performance. While total protein measurements may vary between laboratories due to differences in methods of quantification, the continuous monitoring of total protein appears to be a valuable approach for identifying patients at high risk for subsequent thrombocytopenia, as the time‐dependent variations in total protein were associated with subsequent fluctuations in platelet counts.

Several mathematical models of drug‐induced myelosuppression and short‐term platelet reconstitution in patients without allo‐HCT have been previously published.^33^, ^34^, ^35^, ^36^, ^37^, ^38^, ^42^ Kheifetz *et al*.^43^ presented a mechanistic model that was recently employed to predict thrombocytopenia at subsequent chemotherapy cycles.^44^ Solans *et al*.^45^ introduced a model of neutrophil recovery in pediatric patients with allo‐HCT, incorporating concentration measurements of the conditioning drugs. Similar to our approach, this model was derived from an established myelosuppression model,^18^ with various transplantation effects influencing the proliferation and transition rates, the feedback from blood cells to proliferating HSCs, and the number of proliferating cells post‐HCT. The presented model distinguishes itself by being developed and validated on the basis of real‐world data from a comprehensive cohort of 1949 adult patients undergoing allo‐HCT. Moreover, the model offers insights into platelet count predictions for 180 days post‐HCT, starting from day +7. Model robustness was demonstrated by its successful validation against both internal and external datasets.

However, this study has limitations. Platelet transfusion data for individual patients was missing and we employed an empirical threshold of 12 × 10^9^/L for the imputation of platelet transfusions, based on the observed clinical practice and improvements in model fit. While this approach was inevitable, a uniform threshold across all patients may not accurately reflect the clinical decision‐making for individual patients. Furthermore, conditioning was implemented into the model without consideration of pharmacokinetic models of fludarabine and ATG,^46^, ^47^, ^48^ as *in vivo* concentration measurements were not available and a combination of drugs was used for conditioning in most patients in our datasets. The feedback from blood cells to HSCs was implemented in the graft model, based on the assumption that only donor‐derived HSCs are present after allo‐HCT, even though patient‐derived HSCs could remain after reduced intensity conditioning. The VPC results indicate that the final model underpredicts platelet counts at the 5th percentile early after allo‐HCT. Future model refinements may involve more complex HCT‐effects or feedback mechanisms, which would require detailed data on treatment regimens and clinical interventions for each patient. Furthermore, there is a potential for bias in the model parameters due to the reliance on retrospective data. Incorporating contemporary data in future adjustments could help refine these parameters, particularly for factors like the HCT‐effect, which may be influenced by ongoing advancements in antineoplastic therapies and quality standards. Moreover, the presented model involves numerous model parameters. Nevertheless, all model parameters could be precisely estimated, and the predictive performance was internally and externally validated.

We acknowledge that conditions leading to protein loss, such as veno‐occlusive disease or transplant‐associated thrombotic microangiopathy, which could not be evaluated due to data limitations, may confound the effects of total protein on post‐HCT platelet kinetics. We thus considered acute GvHD as a covariate due to its potential for protein loss via gastrointestinal or skin involvement, however, conducting an independent analysis of the effects of total protein and acute GvHD was constrained by the risk of introducing collider bias.

In conclusion, these findings strengthen the significance of thrombocytopenia as an indicator for the individual outcome after allo‐HCT. The presented model and exhaustive covariate analysis on this comprehensive real‐world dataset highlights the importance of early platelet counts and total protein as valuable determinants for post‐HCT platelet reconstitution. A web‐based model demonstrator provides the basis for testing clinical implementations and to confirming these findings in future clinical studies. If clinically validated, this model‐based approach for the prediction of individual post‐HCT platelet counts holds the potential to improve the outcome of patients undergoing allo‐HCT.

## FUNDING

The research of the XplOit consortium and the conduct of the prospective XplOit study were funded by grants 031L0027A‐F from the German Federal Ministry of Education and Research (BMBF). T.L. acknowledges support by grant 031L0241 for the BMBF project INSPIRATION (ERACoSysMed) and grant 101057639 from the European Union (Horizon Europe) for the project SafePolyMed. A.T.T. acknowledges support by the Deutsche Forschungsgemeinschaft (DFG) grant FU 356/12‐1.

## CONFLICT OF INTEREST

A.T.T.: Consultancy for Maat Pharma, Biomarin, CSL Behring and Onkowissen. Travel reimbursements by Neovii. All other authors declared no competing interests for this work.

## AUTHOR CONTRIBUTIONS

K.M.G., A.T.T., D.S., and T.L. wrote the manuscript; all authors designed the research; K.M.G., D.S., and T.L. performed the research and analyzed the data.

## Supporting information


Data S1.
