# Supplementary material for: Model‐Based Prediction of Clinically Relevant Thrombocytopenia after Allogeneic Hematopoietic Stem Cell Transplantation
Source: Clin Pharmacol Ther. 2025 Feb 6;117(5):1413–26. doi: 10.1002/cpt.3580 (PMC11993296; doi:10.1002/cpt.3580)
Supplement: Supplementary file 1 — Data S1. [file CPT-117-1413-s001.pdf]

Model-based prediction of clinically-relevant thrombocytopenia after allogeneic hematopoietic stem cell transplantation
Supplementary Materials

Katharina M. Götz [1], Amin T. Turki [2,3], Katharina Och [1], Dominik Selzer [1], Christian Brossette [4], Norbert Graf [4], Jochen Rauch [5], Stefan Theobald [4], Yvonne Braun [4], Kerstin Rohm [5], Gabriele Weiler [5], Simeon Rüdesheim [1,6], Matthias Schwab [6-8], Lisa Eisenberg [9], Nico Pfeifer [9], Stephan Kiefer [5], Ulf Schwarz [10], Claudia Riede [11], Sigrun Smola [12], Dietrich W. Beelen [2], Dominic Kaddu-Mulindwa [13], Jürgen Rissland [12], Jörg Bittenbring [13], Thorsten Lehr [1]

[1] Department of Clinical Pharmacy, Saarland University, Saarbrücken, Germany
[2] Department of Hematology and Stem Cell Transplantation, University Hospital Essen, Essen, Germany
[3] Department of Hematology and Oncology, Marienhospital University Hospital, Ruhr-University Bochum, Bochum, Germany
[4] Department of Pediatric Oncology and Hematology, Saarland University, Homburg, Germany
[5] Department of Biomedical Data & Bioethics, Fraunhofer Institute for Biomedical Engineering (IBMT), Sulzbach, Germany
[6] Dr. Margarete Fischer-Bosch-Institute of Clinical Pharmacology, Stuttgart, Germany
[7] Departments of Clinical Pharmacology, Pharmacy and Biochemistry, University of Tübingen, Tübingen, Germany
[8] Cluster of Excellence iFIT (EXC2180) "Image-guided and Functionally Instructed Tumor Therapies", University of Tübingen, Tübingen, Germany
[9] Department of Computer Science, University of Tübingen, Tübingen, Germany
[10] Institute for Formal Ontology and Medical Information Science (IFOMIS), Saarland University, Saarbrücken, Germany
[11] Averbis GmbH, Freiburg, Germany
[12] Institute of Virology, Saarland University Medical Center, Homburg, Germany
[13] Department of Internal Medicine 1, University Hospital Saarland, Homburg, Germany

Correspondence to:
Professor Thorsten Lehr, PhD, Saarland University, Campus C4 3, 66123 Saarbrücken, Germany.
Email: thorsten.lehr@mx.uni-saarland.de

Table of contents

Supplementary Materials and Methods 3

Dataset preparation 3

Model development 3

Covariate analysis 3

Prediction of individual platelet counts after allo-HCT 4

Cross-validation 4

Software 4

Supplementary References 5

Supplementary Tables 6

Table S1: List of baseline patient characteristics that were examined during pre-selection of covariates including the percentages of missing values. 6

Table S2: List of model parameter-covariate combinations that were tested during covariate analysis including the functional form. 7

Table S3: List of laboratory markers which were tested as time-dependent covariates on the proliferation of hematopoietic stem cells from the graft including the percentages of patients without any measurement of the respective laboratory markers. 8

Table S4: Parameter estimates with relative standard error (RSE%) for the final model. 9

Table S5: Parameter estimates with relative standard error (RSE%) for the final model (training dataset) and each of the five-fold subsets (cross validation). 10

Supplementary Figures 11

Figure S1. Scatterplots of empirical Bayesian estimates of the baseline platelet count post-HCT and continuous covariates that were available for more than 80% of the patients in the retrospective cohort. 11

Figure S2. Box-and-whisker plots of empirical Bayesian estimates of the baseline platelet count post-HCT and categorical covariates that were available for more than 80% of the patients in the retrospective cohort. 12

Figure S3. Scatterplots of empirical Bayesian estimates of the mean maturation time post-HCT and continuous covariates that were available for more than 80% of the patients in the retrospective cohort. 13

Figure S4. Box-and-whisker plots of empirical Bayesian estimates of the mean maturation time post-HCT and categorical covariates that were available for more than 80% of the patients in the retrospective cohort. 14

Figure S5. Scatterplots of empirical Bayesian estimates of the HCT-effect and continuous covariates that were available for more than 80% of the patients in the retrospective cohort. 15

Figure S6. Box-and-whisker plots of empirical Bayesian estimates of the HCT-effect and categorical covariates that were available for more than 80% of the patients in the retrospective cohort. 16

Figure S7. Scatterplots of empirical Bayesian estimates of the feedback parameter Gamma and continuous covariates that were available for more than 80% of the patients in the retrospective cohort. 17

Figure S8. Box-and-whisker plots of empirical Bayesian estimates of the feedback parameter Gamma and categorical covariates that were available for more than 80% of the patients in the retrospective cohort. 18

Figure S9. Randomly selected observed platelet–time profiles of 24 patients with a minimum observation period of 80 days post-HCT from the training dataset are shown. 19

Figure S10. Goodness-of-fit plots for the final model. 20

Figure S11. Randomly selected model predicted platelet–time profiles of 24 patients with a minimum observation period of 80 days post-HCT from the training dataset are shown. 21

Figure S12. Simulation of a platelet count-time profile for a typical patient after allo-HCT including the simulated patient- and donor-derived hematopoietic stem cells and blood cells which derive from the patient and the graft. 22

Figure S13. Predictive performance of the final model during model evaluation (five-fold cross-validation). 23

Figure S14. Screencaptures of the “Precise Platelets” application input panels. 24

Figure S15. Screencaptures of the “Precise Platelets” application output. 25

NONMEM control stream (NM-TRAN) code 26

Supplementary Materials and Methods

Dataset preparation

We collected patient and donor demographics, transplantation characteristics, complications after allogeneic hematopoietic stem cell transplantation (allo-HCT), and time-dependent laboratory data as well as viral kinetics in a structured data format. Details on the conditioning regimen were extracted from unstructured medical reports using the Health Discovery software platform (AVERBIS, Freiburg, Germany).

We excluded patients with the following characteristics from this analysis: no information on the conditioning regimen (n=12, retrospective cohort; n=3, prospective cohort), only one platelet measurement during conditioning and only one platelet measurement in week one or week two after allo-HCT (n=106, retrospective cohort; n=16, prospective cohort), and death before day +28 post-HCT (n=26, retrospective cohort; n=5, prospective cohort). We randomly allocated retrospectively collected data into a training dataset, consisting of two-thirds of the patients (n=1048), for model development, and a test dataset, consisting of one-third of the patients (n=518), for internal model validation. Prospectively collected data from 383 patients were used as a validation dataset for external model validation. In total, model development, internal and external validations comprised 80353, 39765 and 26935 platelet measurements.

Model development

In our adaptation of an established myelosuppression model,[1] cell maturation is characterized by the transition of hematopoietic stem cells (HSCs) through three differentiation stages in the bone marrow before entering the peripheral blood, where circulating blood cells are removed from the body. In absence of relevant data for the estimation of distinct rates for proliferation, differentiation, and elimination, these processes were represented by an estimated mean maturation time (MMT). We tested several model extensions to reflect the transplantation of donor-derived HSCs, including (1) re-estimation of model parameters for time after allo-HCT, (2) re-estimation of model parameters for time after a change point reflecting the time of platelet engraftment, (3) a time-dependent feedback mechanism stimulating the proliferation of donor-derived HSCs with a time delay, (4) a time-dependent cell maturation time to delay the appearance of platelets after allo-HCT, (5) dosing of virtual stem cells to constitute the proliferating HSCs of a new hematopoietic system post-HCT, and (6) the incorporation of a transplantation effect to be tested on different model parameters. We also tested combinations of different model extensions.

The change of kinetics over time was described via ordinary differential equations, solved numerically with LSODA (subroutine ADVAN 13 in NONMEM®).[2] The model parameters for the population (Θ), inter-patient variability (η) and residual variability were estimated via stochastic approximation expectation maximization (SAEM).[3] Of note, during the initial stages of model development, we utilized first-order conditional estimation with eta-epsilon interaction (FOCEI) for rapid initial model space exploration due to beneficial model run times in contrast to SAEM. The objective function value (OFV; maximum likelihood estimation of fixed and random effects)[4] was obtained by post-hoc importance sampling. Inter-patient variability was modelled using exponential random effects, which were initially included on all model parameters. Subsequently, we removed random effects that were estimated to be near zero. During graphical evaluation, if the η-distribution was found to be skewed, we applied a Box-Cox transformation to the inter-patient variability and estimated optimal transformation parameters. The complete variance-covariance matrix was assessed, and covariance terms estimated to be close to zero were subsequently removed. Residual variability was tested using various models, including proportional, additive, and combined proportional and additive error models. For nested models, a decrease of the OFV (dOFV) exceeding 10.83 points, corresponding to a p-value of approximately 0.001 (likelihood ratio test), was considered significant for the inclusion of one additional parameter (df=1) into the model. The conservative selection threshold for dOFV was set to mitigate overfitting, considering the cardinality of the training dataset (number of individuals and data points) as well as the variability in the OFV for repeated runs of a model due to the stochastic nature of the SAEM algorithm.

Covariate analysis

We investigated static variables and time-dependent laboratory markers that were available for more than 80% of the patients in the retrospective cohort. For completely missing continuous and categorical covariates, we imputed values using the median and mode of the respective patient cohort, respectively. Unknown pre-HCT values of time-dependent laboratory markers were imputed with the subsequently observed values, and missing post-HCT values were imputed with the last observed values.

We performed covariate analysis on the base model, testing categorical variables using a proportional shift model with an estimated coefficient parameter reflecting the proportional increase or decrease of a model parameter for one group over a reference group. For continuous variables, we used a power model, centering the variable around the median value and estimating an exponent. Variables explaining inter-patient variability of a model parameter by more than 5% were considered significant and incorporated into the final model.

The final model was utilized to simulate the effects of covariates on the platelet-time course following allo-HCT. For this, separate simulations were conducted for each included covariate. Continuous covariates were set to the median as well as to the 10th and 90th percentile values, while categorical covariates were assigned an effect value and a no-effect value. Unmodulated continuous and categorical covariates were set to median and no-effect values.

Prediction of individual platelet counts after allo-HCT

The final model and early patient data were used to predict individual platelet-time profiles for up to 180 days post-HCT using Bayesian forecasting with model parameters estimated from the training dataset as priors. To compute the 95% prediction intervals, we performed 1000 simulations per individual using the random effect point estimates and their covariance matrix from the Maximum a Posteriori (MAP) estimation step. From these simulations, we calculated the median, 2.5th, and 97.5th percentiles for all model outputs.
We investigated the predictive performance at four different time points (days +7, +14, +21, +28) using quantitative and graphical criteria. First, observed platelet counts were categorised as grade 2 thrombocytopenia or no grade 2 thrombocytopenia according to the mean value of the last twelve platelet measurements in the first 180 days after allo-HCT, with mean values $<75 \times 10^9$/L defined as grade 2 thrombocytopenia. We then compared model-predicted platelet counts, with a 5% tolerance for prediction variability, against a range of cut-off values from 10×10^9/L to 384×10^9/L (simulation range). For each cut-off, we calculated true/false positives/negatives to determine sensitivity and specificity, and generated Receiver Operating Characteristic (ROC) curves. The area under the ROC curve (AUROC) quantified the accuracy, complemented by a bootstrap analysis (n=1000) to derive 95% confidence intervals for AUROC values and the ROC curve. Of note, model predictions were compared to cut-off values (simulation range) considering a 5% variation to accommodate the coefficient of variation in electrical impedance methods used for determining platelet counts. Ensuring that the absolute variation was adjusted according to the value of the respective cut-off, this approach allowed for a proportionally smaller variation in low cut-offs, which are indicative of thrombocytopenia.
Additionally, mean and 10th/90th percentile values of observed and predicted counts were analysed for a comprehensive graphical assessment.

Cross-validation

To investigate potential biases in the estimated model parameters, overfitting, and model robustness, we performed five-fold cross-validation. Therefore, the retrospective cohort (n=1566) was randomly divided into five evenly distributed subsets. Then, the data from four subsets was repeatedly used for the estimation of model parameters. The estimated model parameters of all five subsets were compared to the model parameters derived from the training dataset (n=1048). Next, the estimated parameters were used to predict the data of the retained subset and the predictive performance was assessed for all five subsets. Here, we investigated the predictive performance for each subset at day +28 using ROC curves and AUROC values.

Software

The Statistical Software R (3.6.3, R Foundation for Statistical Computing, Vienna, Austria) was used for dataset preparation, statistical analyses, and graphical presentation. The following R packages were used for analysis and plot generation:
- boot (version 1.3.28.1; Canty A, Ripley BD, 2022)
- dplyr (version 1.1.2; Wickham H et al., 2023)
- ggplot2 (version 3.5.0; Wickham H, 2016)
- ggpubr (version 0.6.0; Kassambara A, 2023)
- gridExtra (version 2.3; Auguie B, 2017)
- lubridate (version 1.9.2; Grolemund G, Wickham H, 2011)
- magrittr (version 2.0.3; Bache S, Wickham H, 2022)
- MASS (version 7.3.60.0.1; Venables WN, Ripley BD, 2002)
- pammtools (version 0.5.93; Bender A, Scheipl F, 2018)
- pracma (version 2.4.4; Borchers H, 2023)
- purrr (version 1.0.1; Wickham H, Henry L, 2023)
- survminer (version 0.4.9; Kassambara A et al., 2021)
- survival (version 3.5.5; Therneau T, 2023)
- tidyr (version 1.3.0; Wickham H et al., 2023)

Supplementary References

1. Friberg LE, Henningsson A, Maas H, Nguyen L, Karlsson MO. Model of chemotherapy-induced myelosuppression with parameter consistency across drugs. *J Clin Oncol*. 2002;20(24):4713-4721. doi:10.1200/JCO.2002.02.140
2. Petzold L. Automatic selection of methods for solving stiff anf nonstiff systems of ODEs.pdf. *SIAM J Sci Stat Comput*. 1983;4(1):136-148.
3. Delyon B, Lavielle M, Moulines E. Convergence of a stochastic approximation version of the EM algorithm. *Ann Stat*. 1999;27(1):94-128. doi:10.1214/aos/1018031103
4. Bae KS, Yim DS. R-based reproduction of the estimation process hidden behind NONMEM® part 2: First-order conditional estimation. *Transl Clin Pharmacol*. 2016;24(4):161-168. doi:10.12793/tcp.2016.24.4.161

Supplementary Tables

Table S1: List of baseline patient characteristics that were examined during pre-selection of covariates including the percentages of missing values.

	**Retrospective cohort** (n = 1566)	**Prospective cohort** (n = 383)
**Variable**	**Missingness, %**	**Missingness, %**
Acute GvHD	0.00	0.00
Acute GvHD grades	0.00	0.00
Acute GvHD onset	0.77	0.00
Anti-thymocyte globulin (ATG)	1.15	1.57
Anti-thymocyte globulin (ATG), dose	1.15	1.57
Baseline immunosuppression	0.00	0.00
Blood group donor	0.06	1.04
Blood group patient	0.19	1.57
CD34+ stem cell dose	12.77	12.01
CMV serostatus donor	1.21	1.57
CMV serostatus patient	1.28	1.57
Chronic GvHD	0.00	0.00
Chronic GvHD stadium	0.00	0.00
Conditioning intensity, MAC/RIC	0.00	0.00
Conditioning regimen	0.57	0.52
Death	0.00	0.00
Death, days between allo-HCT and death	0.00	0.00
Diagnosis	0.00	0.00
Disease stage before allo-HCT	13.28	23.5
Donor type	0.00	0.00
Donor age	0.19	0.00
Donor relationship	0.00	0.00
Donor sex	0.00	0.00
HLA matching, 10/10 vs. others	0.00	0.00
Months between diagnosis and allo-HCT	0.13	0.78
Patient age	0.00	0.00
Patient sex	0.00	0.00
Relapse	0.00	0.00
Relapse, days between allo-HCT and relapse	0.00	0.00
Sex mismatch	0.00	0.00
Stem cell source	0.06	0.00
Total body irradiation (TBI)	0.00	0.00

Table S2: List of model parameter-covariate combinations that were tested during covariate analysis including the functional form.

Model parameter	Covariate	Type of covariate	Category tested	Functional form
Patient model				
Baseline platelet count pre-HCT	Diagnosis	categorical	Myelodysplastic syndromes	Additive Shift
Baseline platelet count pre-HCT	Diagnosis	categorical	Myelomonocytic leukemia	Additive Shift
Baseline platelet count pre-HCT	Diagnosis	categorical	Other hematologic malignancies	Additive Shift
Baseline platelet count pre-HCT	Disease stage	categorical	Advanced	Additive Shift
Baseline platelet count pre-HCT	Subgroup [a]	categorical	Subgroups with high or low cell counts	Additive Shift
Drug effect	Conditioning regimen	categorical	Fludarabin	Additive Shift
Drug effect	Conditioning regimen	categorical	Etoposid	Additive Shift
Drug effect	Conditioning regimen	categorical	Cyclophosphamid	Additive Shift
Drug effect	Conditioning regimen	categorical	Melphalan	Additive Shift
Drug effect	Conditioning treatment	continuous	Duration, days	Linear
Drug effect	Diagnosis	categorical	Other hematologic malignancies	Additive Shift
Graft model				
Baseline platelet count post-HCT	Anti-thymocyte globulin (ATG)	categorical	Yes	Additive Shift
Baseline platelet count post-HCT	Acute GvHD	categorical	Grades 2-4	Additive Shift
Baseline platelet count post-HCT	Acute GvHD	categorical	Grades ≥ 3	Additive Shift
Baseline platelet count post-HCT	GvHD liver	categorical	Grades 2-4	Additive Shift
Baseline platelet count post-HCT	Diagnosis	categorical	Chronic lymphocytic leukemia	Additive Shift
Baseline platelet count post-HCT	Diagnosis	categorical	Myeloproliferative disorders	Additive Shift
Baseline platelet count post-HCT	HLA matching	categorical	10/10 match	Additive Shift
Baseline platelet count post-HCT	Donor type	categorical	Matched related donor (MRD)	Additive Shift
Baseline platelet count post-HCT	Donor type	categorical	Mismatched related donor (MMRD)	Additive Shift
Baseline platelet count post-HCT	Number of platelet transfusions	continuous		Linear
Baseline platelet count post-HCT	Number of platelet transfusions	categorical	> 5	Additive Shift
Baseline platelet count post-HCT	Stem cell source	categorical	Bone marrow	Additive Shift
Baseline platelet count post-HCT	Stem cell source	categorical	Bone marrow + peripheral blood stem cells	Additive Shift
Baseline platelet count post-HCT	Subgroup [a]	categorical	Subgroups with high or low cell counts	Additive Shift
Baseline platelet count post-HCT	CD34+ stem cell dose	continuous		Power
Baseline platelet count post-HCT	CD34+ stem cell dose	categorical	$< 5 \times 10^6$ cells	Additive Shift
Mean maturation time post-HCT	Anti-thymocyte globulin (ATG)	categorical	Yes	Additive Shift
Mean maturation time post-HCT	Total protein	time-dependent		Power
γ, feedback mechanism parameter	Anti-thymocyte globulin (ATG)	categorical	Yes	Additive Shift
γ, feedback mechanism parameter	Total protein	time-dependent		Power
HCT-effect	Anti-thymocyte globulin (ATG)	categorical	Yes	Additive Shift
HCT-effect	Conditioning regimen	categorical	Fludarabin	Additive Shift
HCT-effect	Stem cell source	categorical	Bone marrow	Additive Shift
HCT-effect	Donor	categorical	Matched related donor	Additive Shift
HCT-effect elimination	Anti-thymocyte globulin (ATG)	categorical	Yes	Additive Shift
HCT-effect elimination	Conditioning regimen	categorical	Cyclophosphamid	Additive Shift
HCT-effect elimination	Conditioning regimen	categorical	Etoposid	Additive Shift
HCT-effect elimination	Conditioning regimen	categorical	Melphalan	Additive Shift
HCT-effect elimination	Stem cell source	categorical	Bone marrow	Additive Shift
HCT-effect elimination	Stem cell source	categorical	Peripheral blood stem cells	Additive Shift
HCT-effect elimination	Stem cell source	categorical	Bone marrow + peripheral blood stem cells	Additive Shift
Apheresis cells	Donor relation	categorical	Related	Additive Shift
Apheresis cells	Donor	categorical	Matched related donor (MRD)	Additive Shift

[a] Two model parameters were estimated for different patient subgroups using a NONMEM mixture model.

Table S3: List of laboratory markers which were tested as time-dependent covariates on the proliferation of hematopoietic stem cells from the graft including the percentages of patients without any measurement of the respective laboratory markers.

		**Retrospective cohort** (n = 1566)	**Prospective cohort** (n = 383)
**Variable**	**Unit**	**Missingness [a], %**	**Missingness [a], %**
Alanine transaminase (ALT)	U/L	0.00	0.00
Albumin	g/dL	2.17	0.78
Alkaline phosphatase (AP)	U/L	0.00	0.00
Aspartate aminotransferase (AST)	U/L	0.00	0.00
Basophils	$\times 10^9$/L	0.00	0.00
Bilirubin	mg/dL	0.00	0.00
C-reactive protein (CRP)	mg/dL	0.00	0.00
Gamma-glutamyl transferase (GGT)	U/L	0.00	0.00
Hemoglobin	g/dL	0.00	0.00
Lactate dehydrogenase (LDH)	U/L	0.00	0.00
Lymphocytes	$\times 10^9$/L	0.00	0.00
Mean corpuscular hemoglobin (MCH)	pg	0.00	0.00
Mean corpuscular volume (MCV)	fL	0.00	0.00
Mean platelet volume (MPV)	fL	3.9	0.26
Neutrophils	$\times 10^9$/L	0.00	0.00
Platelets	$\times 10^9$/L	0.00	0.00
Red blood cells	$\times 10^{12}$/L	0.00	0.00
Red cell distribution width (RDW)	fL	0.00	0.00
Reticulocyte hemoglobin equivalent (Ret-He)	pg	3.58	4.18
Reticulocytes	$\times 10^9$/L	0.06	2.61
Total cholesterol	mg/dL	0.00	0.00
Total protein	g/dL	0.00	0.00
Urea	mg/dL	0.00	0.00
Uric acid	mg/dL	0.00	0.00
White blood cells	$\times 10^9$/L	0.00	0.00

[a] Patients without any measurement of the respective laboratory marker throughout the whole observation period.

Table S4: Parameter estimates with relative standard error (RSE%) for the final model.

**Model Parameter**	**Population Estimate (RSE%)**	**Inter-patient variability (RSE%)**	**Description**
Patient model			
Baseline, × 10^9/L	90.5 (4)	115.0 [a] (5)	Baseline platelet count pre-HCT
MMT_{PAT}, days	6.11 (3)	-	Mean maturation time pre-HCT
Drug effect	0.945 (16)	152.3 [a] (38)	Inhibition effect of the conditioning treatment
ATG effect	0.00212 (5)	-	Prohibition effect of ATG
K_{INT}, days^{-1}	0.693 (7)	-	ATG elimination rate constant
Apheresis cells (Θ_{AC}), × 10^9/L	20.1 (28)	289.7 [a] (25)	Increase in platelet counts on day +1
Platelet concentrates, × 10^9/L	10.6 (5)	67.9 [a] (17)	Increase in platelet counts after administration of platelet concentrates
Graft model			
TTBM, days	1.54 (15)	-	Time to bone marrow
Graft cells (Θ_{Graft}), × 10^9/L	72.4 (9)	82.9 [a] (9)	Baseline platelet count post-HCT
MMT_{GT}, days	6.99 (8)	90.9 [a] (10)	Mean maturation time post-HCT
γ (Θ_γ)	0.192 (7)	48.7 [a] (12)	Feedback mechanism parameter
HCT-effect	4.22 (29)	87.1 [a] (16)	Prohibition effect of transplantation
K_{EL} (Θ_{KEL}), days^{-1}	0.353 (12)	-	HCT-effect elimination rate constant
Shape Graft cells (Θ_{SHPGT})	-0.484 (31)	-	ETA distribution shape for graft cells
Shape γ (Θ_{SHPGA})	-1.28 (3)	-	ETA distribution shape for γ
Covariates			
Kinship ($\Theta_{Kinship}$)	2.75 (40)		Donor relation parameter for the increase in platelet counts on day +1
Total protein ($\Theta_{Protein}$)	0.197 (9)		Total protein parameter for donor-derived HSC proliferation
Residual error			
Proportional, %CV	34.4 (2)		
Additive, × 10^9/L	3.98 (6)		
Correlations			
Baseline, Drug effect	0.589		Correlation of platelet count pre-HCT and prohibition effect of the conditioning treatment
Graft cells, MTT_{GT}	-0.291		Correlation of platelet count post-HCT and mean maturation time post-HCT

Abbreviations: ATG, anti-thymocyte globulin; K_{INT}, ATG elimination rate constant; K_{EL}, HCT-effect elimination rate constant; γ, feedback mechanism parameter; GT, graft; MMT, mean maturation time; PAT, patient; TTBM, time to bone marrow.

$$\text{Apheresis cells} = \Theta_{AC} \times \left(1 + \Theta_{Kinship} \times \text{Kinship}\right) \times \exp\left(\eta_{\text{Apheresis cells}}\right).$$

$$\text{Graft cells} = \Theta_{Graft} \times \exp\left(\frac{\exp\left(\eta_{Graft}\right)^{\Theta_{SHPGT}} - 1}{\theta_{SHPGT}}\right).$$

$$\gamma = \Theta_\gamma \times \exp\left(\frac{\exp\left(\eta_{GA}\right)^{\Theta_{SHPGA}} - 1}{\theta_{SHPGA}}\right).$$

η = inter-patient variation.

[a] inter-patient variability calculated from $\sqrt{\exp(\omega^2) - 1}$. ω = variance.

Table S5: Parameter estimates with relative standard error (RSE%) for the final model (training dataset) and each of the five-fold subsets (cross validation).

	**Training Dataset (n=1048)**	**Subset 1 (n=1255)**	**Subset 2 (n=1249)**	**Subset 3 (n=1257)**	**Subset 4 (n=1253)**	**Subset 5 (n=1250)**
Model Parameter	Estimate (RSE%)	Estimate (RSE%)	Estimate (RSE%)	Estimate (RSE%)	Estimate (RSE%)	Estimate (RSE%)
Patient model						
Baseline, × 10^9/L	90.5 (4)	88.8 (3)	90.3 (3)	87.8 (3)	88.1 (3)	88.7 (3)
MMT_{PAT}, days	6.11 (3)	6.03 (0)	6.02 (0)	6.21 (0)	5.99 (0)	6.03 (0)
Drug effect	0.945 (16)	0.854 (4)	0.856 (4)	0.925 (4)	0.865 (4)	0.846 (4)
ATG effect	0.00212 (5)	0.00218 (1)	0.00208 (1)	0.00214 (1)	0.00196 (1)	0.00201 (1)
K_{INT}, days^{-1}	0.693 (7)	0.745 (1)	0.709 (1)	0.711 (1)	0.71 (1)	0.674 (1)
Apheresis cells (Θ_{AC}), × 10^9/L	20.1 (28)	15.8 (9)	17.5 (9)	15.1 (10)	16.3 (9)	17.4 (9)
Platelet concentrates, × 10^9/L	10.6 (5)	11 (4)	10.9 (4)	11 (3)	11 (3)	11 (3)
Graft model						
TTBM, days	1.54 (15)	2.04 (2)	1.69 (2)	1.79 (1)	1.53 (2)	1.64 (2)
Graft cells (Θ_{Graft}), × 10^9/L	72.4 (9)	74.7 (2)	71.9 (2)	75.8 (2)	75.6 (2)	75.5 (2)
MMT_{GT}, days	6.99 (8)	6.45 (2)	6.96 (2)	6.73 (2)	6.74 (2)	6.57 (2)
γ (Θ_γ)	0.192 (7)	0.188 (2)	0.191 (2)	0.192 (2)	0.189 (1)	0.176 (2)
HCT-effect	4.22 (29)	6.5 (4)	4.86 (4)	4.59 (4)	4.35 (4)	5.61 (4)
K_{EL} (Θ_{KEL}), days^{-1}	0.353 (12)	0.381 (1)	0.36 (1)	0.35 (1)	0.358 (1)	0.384 (1)
Shape Graft cells (Θ_{SHPGT})	-0.484 (31)	-0.547 (4)	-0.516 (3)	-0.524 (4)	-0.462 (5)	-0.538 (4)
Shape γ (Θ_{SHPGA})	-1.28 (3)	-1.23 (3)	-1.27 (3)	-1.16 (3)	-1.26 (3)	-1.12 (2)
Covariates						
Kinship ($\Theta_{Kinship}$)	2.75 (40)	2.53 (21)	2.39 (20)	2.6 (22)	2.38 (21)	2.8 (19)
Total protein ($\Theta_{Protein}$)	0.197 (9)	0.21 (1)	0.205 (1)	0.189 (1)	0.204 (1)	0.201 (1)
Inter-patient variability						
Baseline, %CV	115% (5)	114.3% (3)	115.6% (3)	114.3% (3)	116.7% (3)	116.3% (3)
Drug effect, %CV	152.3% (38)	129.9% (3)	130.5% (3)	139.5% (3)	140.5% (3)	128% (3)
Graft cells, %CV	82.9% (9)	82.8% (3)	85.7% (3)	82.5% (2)	81.4% (2)	81.4% (2)
MMT_{GT}, %CV	90.9% (10)	101.3% (2)	101.5% (2)	100% (2)	93.1% (2)	98.3% (2)
HCT-effect, %CV	87.1% (16)	146.9% (2)	136.3% (2)	108.9% (2)	110.6% (1)	131.1% (2)
γ, %CV	48.7% (12)	49.7% (3)	49.2% (3)	50.7% (3)	48% (3)	53.2% (3)
Apheresis cells (Θ_{AC}), %CV	289.7% (25)	393% (3)	336.2% (4)	421% (3)	356.9% (3)	355% (3)
Platelet concentrates, %CV	67.9% (17)	70.3% (3)	70.7% (3)	62.9% (3)	69.1% (3)	63.1% (3)
Correlations						
Baseline, Drug effect	0.589	0.566	0.553	0.564	0.605	0.564
Graft cells, MTT_{GT}	-0.291	-0.292	-0.316	-0.266	-0.258	-0.281
Residual error						
Proportional, %CV	34.4% (2)	34.1% (0)	34.4% (0)	34.3% (0)	34.7% (0)	35.1% (0)
Additive, × 10^9/L	3.98 (6)	3.99 (1)	4.14 (1)	4.01 (1)	3.80 (1)	3.76 (1)

Abbreviations: ATG, anti-thymocyte globulin; K_{INT}, ATG elimination rate constant; K_{EL}, HCT-effect elimination rate constant; γ, feedback mechanism parameter; GT, graft; MMT, mean maturation time; PAT, patient; TTBM, time to bone marrow.

$\text{Apheresis cells} = \Theta_{AC} \times \left(1 + \Theta_{Kinship} \times \text{Kinship}\right) \times \exp\left(\eta_{\text{Apheresis cells}}\right)$.

$\text{Graft cells} = \Theta_{Graft} \times \exp\left(\frac{\exp\left(\eta_{Graft}\right)^{\Theta_{SHPGT}} - 1}{\theta_{SHPGT}}\right)$.

$\gamma = \Theta_\gamma \times \exp\left(\frac{\exp\left(\eta_{GA}\right)^{\Theta_{SHPGA}} - 1}{\theta_{SHPGA}}\right)$.

η = inter-patient variation.

[a] inter-patient variability calculated from $\sqrt{\exp(\omega^2) - 1}$. ω = variance.

Supplementary Figures

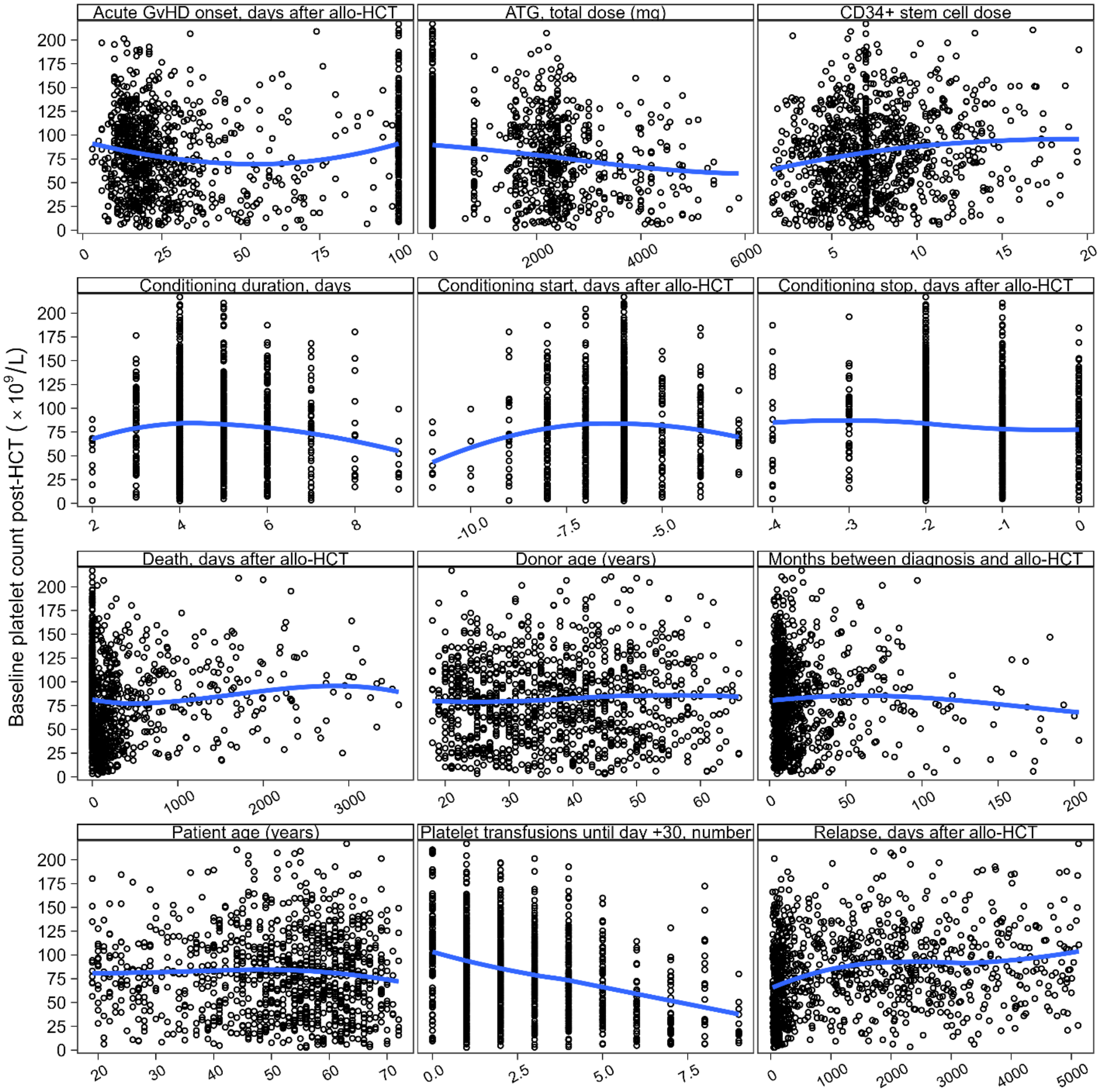

Figure S1. Scatterplots of empirical Bayesian estimates of the baseline platelet count post-HCT and continuous covariates that were available for more than 80% of the patients in the retrospective cohort. The blue lines represent locally estimated scatterplot smoothing (LOESS) regressions. Abbreviations: ATG, anti-thymocyte globulin; GvHD, graft versus host disease.

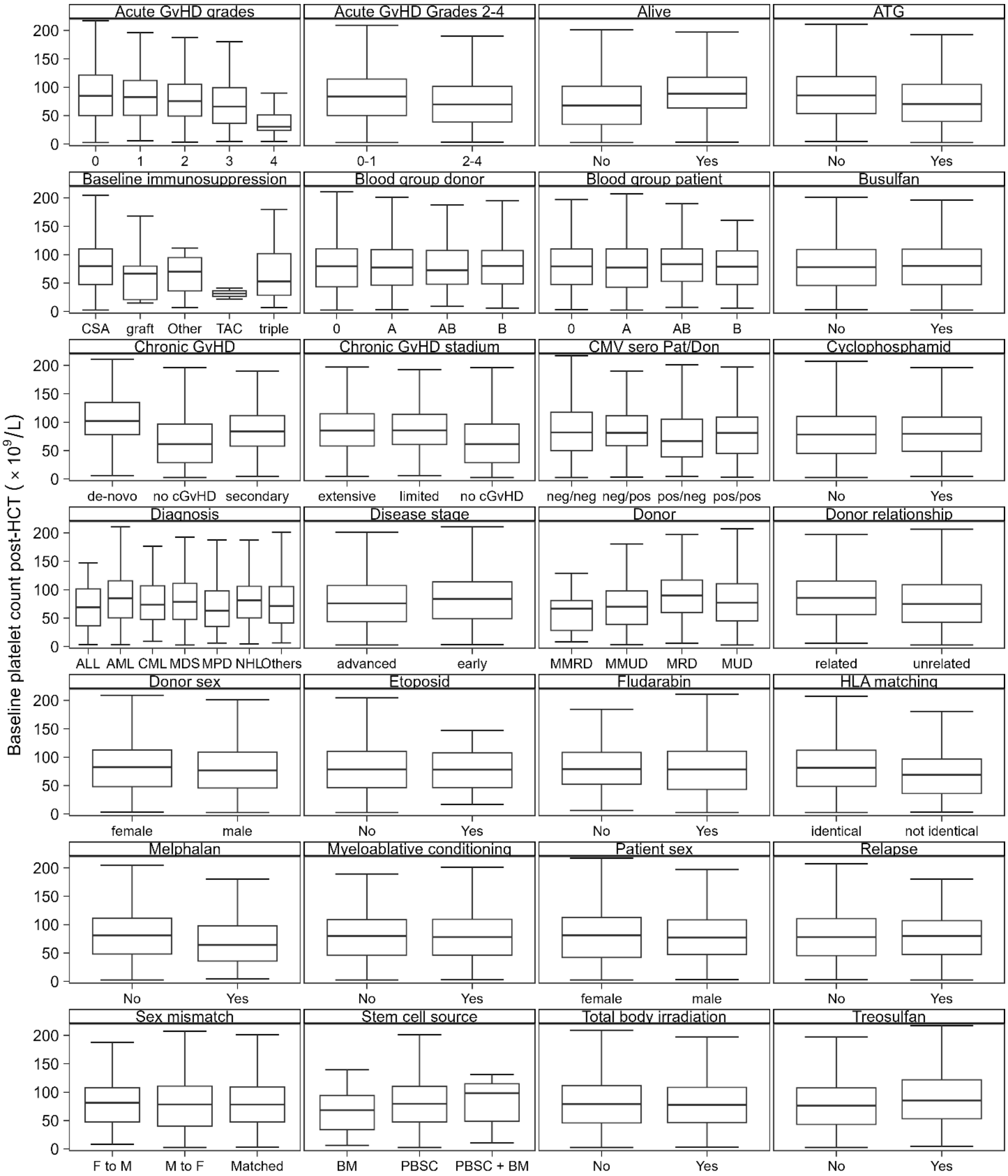

Figure S2. Box-and-whisker plots of empirical Bayesian estimates of the baseline platelet count post-HCT and categorical covariates that were available for more than 80% of the patients in the retrospective cohort. Abbreviations: ALL, Acute lymphoblastic leukemia; AML, Acute myeloid leukemia; ATG, anti-thymocyte globulin; BM, Bone marrow; CML, Chronic myeloid leukemia; CMV, cytomegalovirus; CSA, cyclosporine A; Don, stem cell donor; F, female; GvHD, graft versus host disease; HLA, human leukocyte antigen; neg, negative; M, male; MDS, Myelodysplastic syndromes; MMRD, Mis-matched related donor; MMUD, Mis-matched unrelated donor; MPD; Myeloproliferative Disorder; MRD, Matched related donor; MUD, matched unrelated donor; NHL, Non-Hodgkin's lymphoma; Pat, patient; PBSC, peripheral blood stem cell; pos, positive; TAC, tacrolimus.

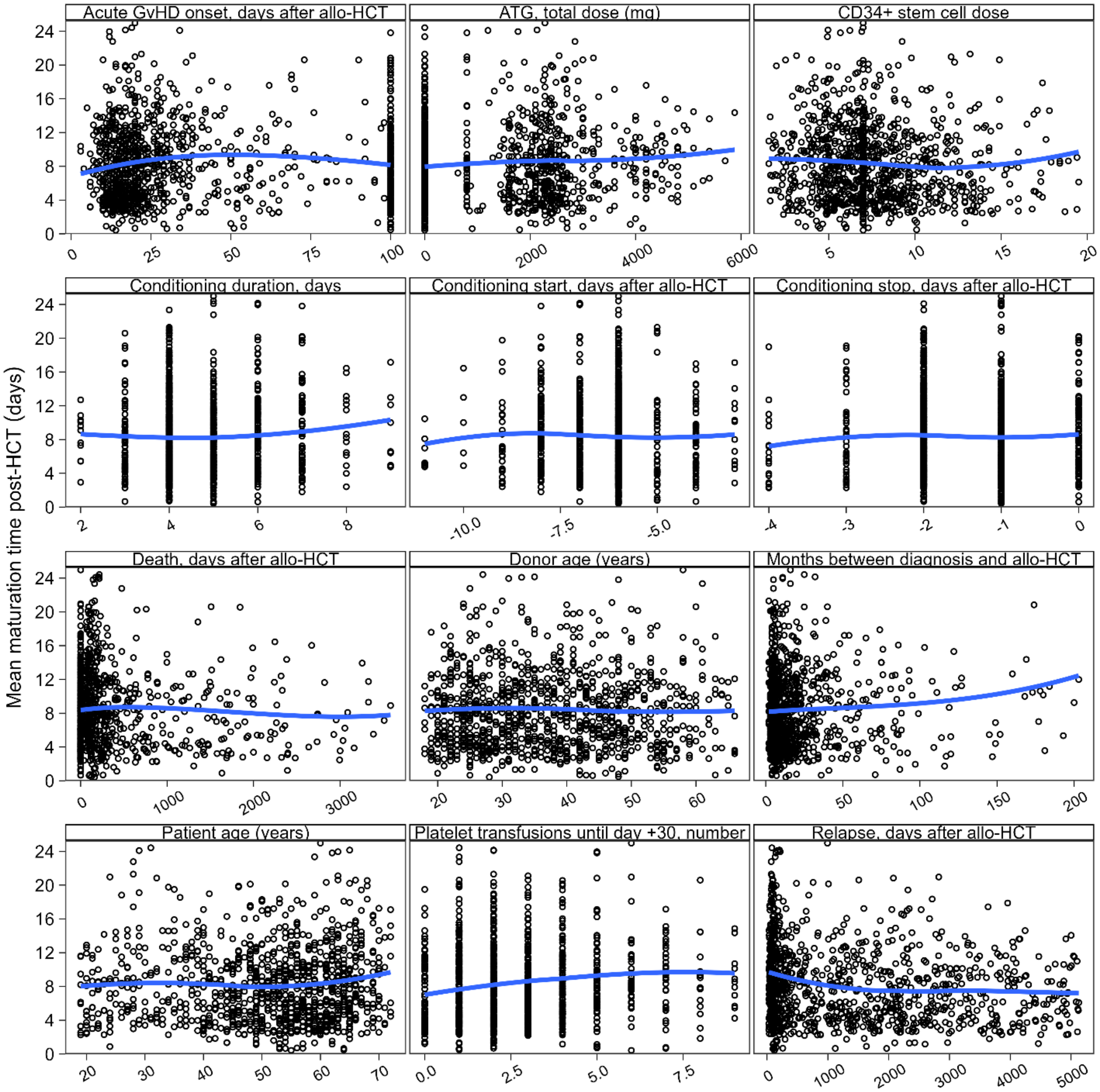

Figure S3. Scatterplots of empirical Bayesian estimates of the mean maturation time post-HCT and continuous covariates that were available for more than 80% of the patients in the retrospective cohort. The blue lines represent locally estimated scatterplot smoothing (LOESS) regressions. Abbreviations: ATG, anti-thymocyte globulin; GvHD, graft versus host disease.

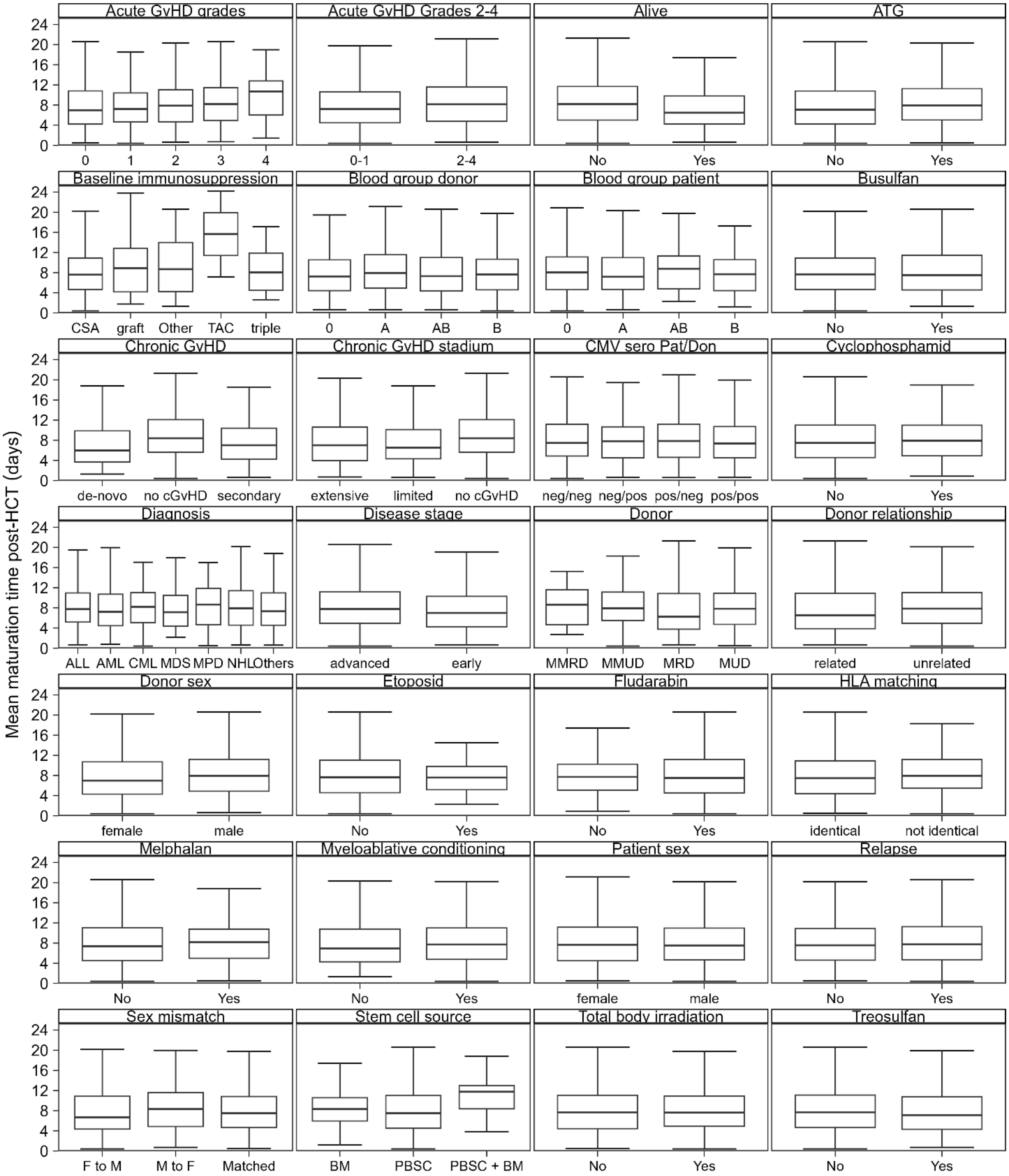

Figure S4. Box-and-whisker plots of empirical Bayesian estimates of the mean maturation time post-HCT and categorical covariates that were available for more than 80% of the patients in the retrospective cohort. Abbreviations: ALL, Acute lymphoblastic leukemia; AML, Acute myeloid leukemia; ATG, anti-thymocyte globulin; BM, Bone marrow; CML, Chronic myeloid leukemia; CMV, cytomegalovirus; CSA, cyclosporine A; Don, stem cell donor; F, female; GvHD, graft versus host disease; HLA, human leukocyte antigen; neg, negative; M, male; MDS, Myelodysplastic syndromes; MMRD, Mis-matched related donor; MMUD, Mis-matched unrelated donor; MPD; Myeloproliferative Disorder; MRD, Matched related donor; MUD, matched unrelated donor; NHL, Non-Hodgkin's lymphoma; Pat, patient; PBSC, peripheral blood stem cell; pos, positive; TAC, tacrolimus.

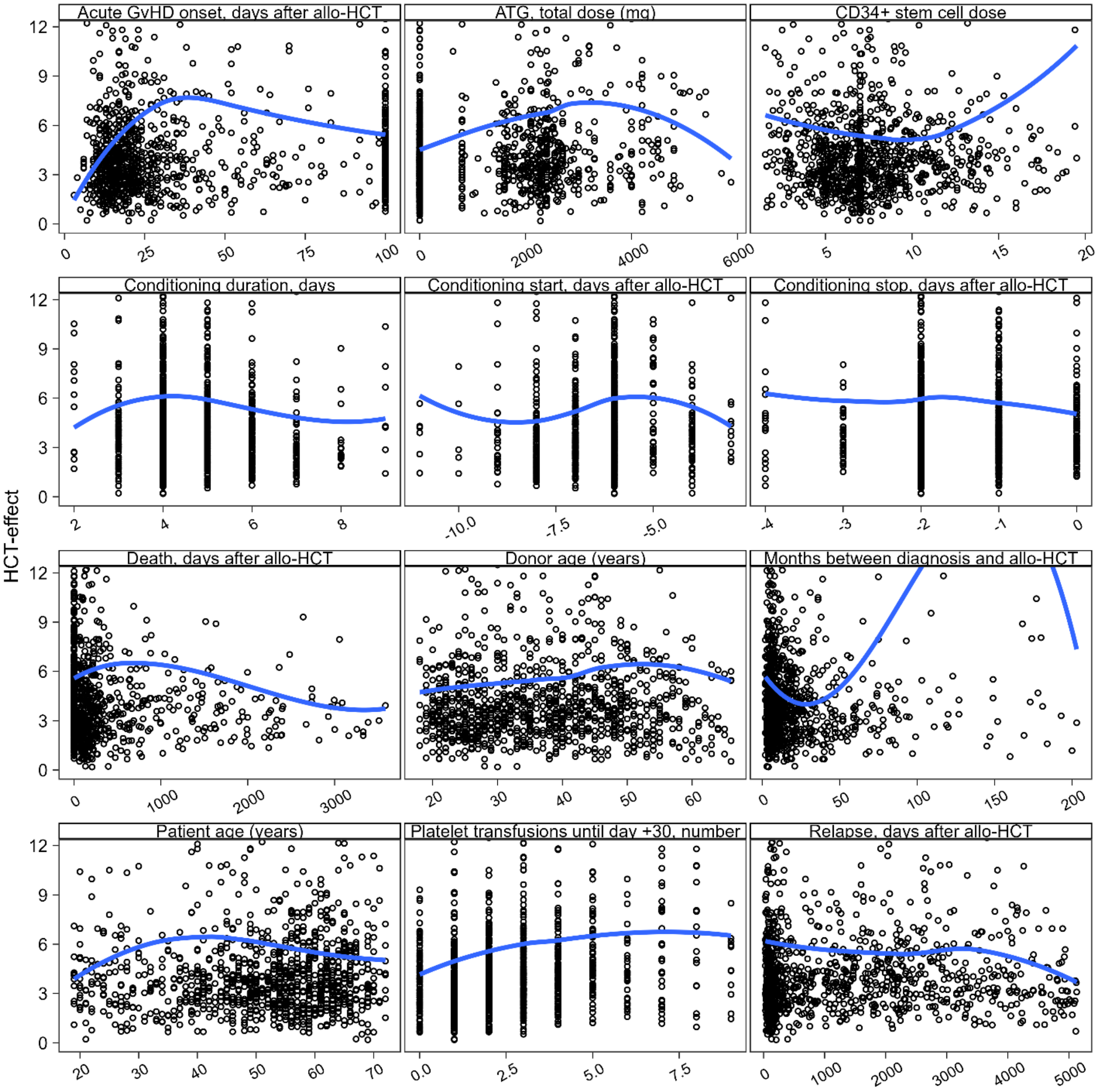

Figure S5. Scatterplots of empirical Bayesian estimates of the HCT-effect and continuous covariates that were available for more than 80% of the patients in the retrospective cohort. The blue lines represent scatterplot smoothing (LOESS) regressions. Abbreviations: ATG, anti-thymocyte globulin; GvHD, graft versus host disease.

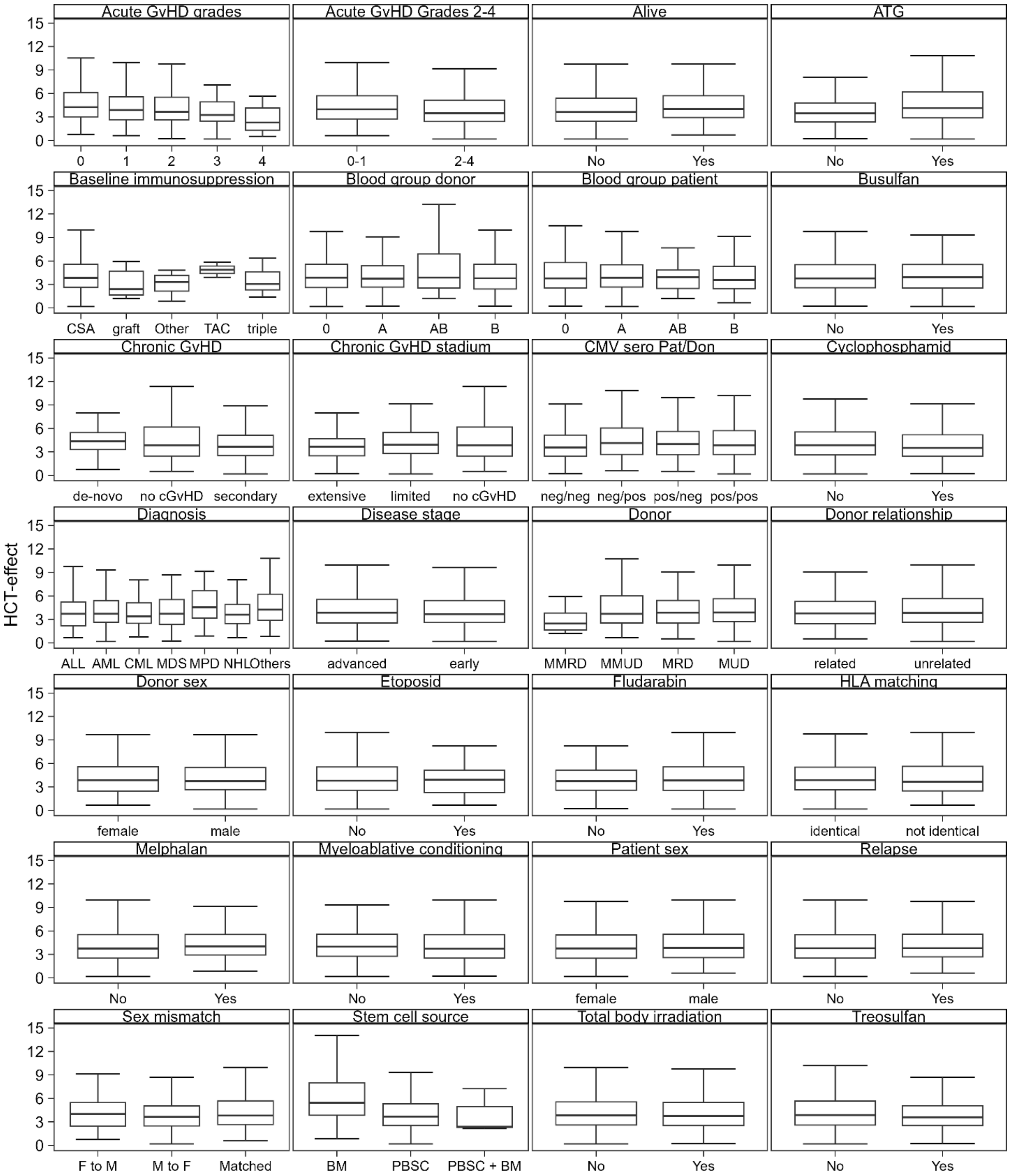

Figure S6. Box-and-whisker plots of empirical Bayesian estimates of the HCT-effect and categorical covariates that were available for more than 80% of the patients in the retrospective cohort. Abbreviations: ALL, Acute lymphoblastic leukemia; AML, Acute myeloid leukemia; ATG, anti-thymocyte globulin; BM, Bone marrow; CML, Chronic myeloid leukemia; CMV, cytomegalovirus; CSA, cyclosporine A; Don, stem cell donor; F, female; GvHD, graft versus host disease; HLA, human leukocyte antigen; neg, negative; M, male; MDS, Myelodysplastic syndromes; MMRD, Mis-matched related donor; MMUD, Mis-matched unrelated donor; MPD; Myeloproliferative Disorder; MRD, Matched related donor; MUD, matched unrelated donor; NHL, Non-Hodgkin's lymphoma; Pat, patient; PBSC, peripheral blood stem cell; pos, positive; TAC, tacrolimus.

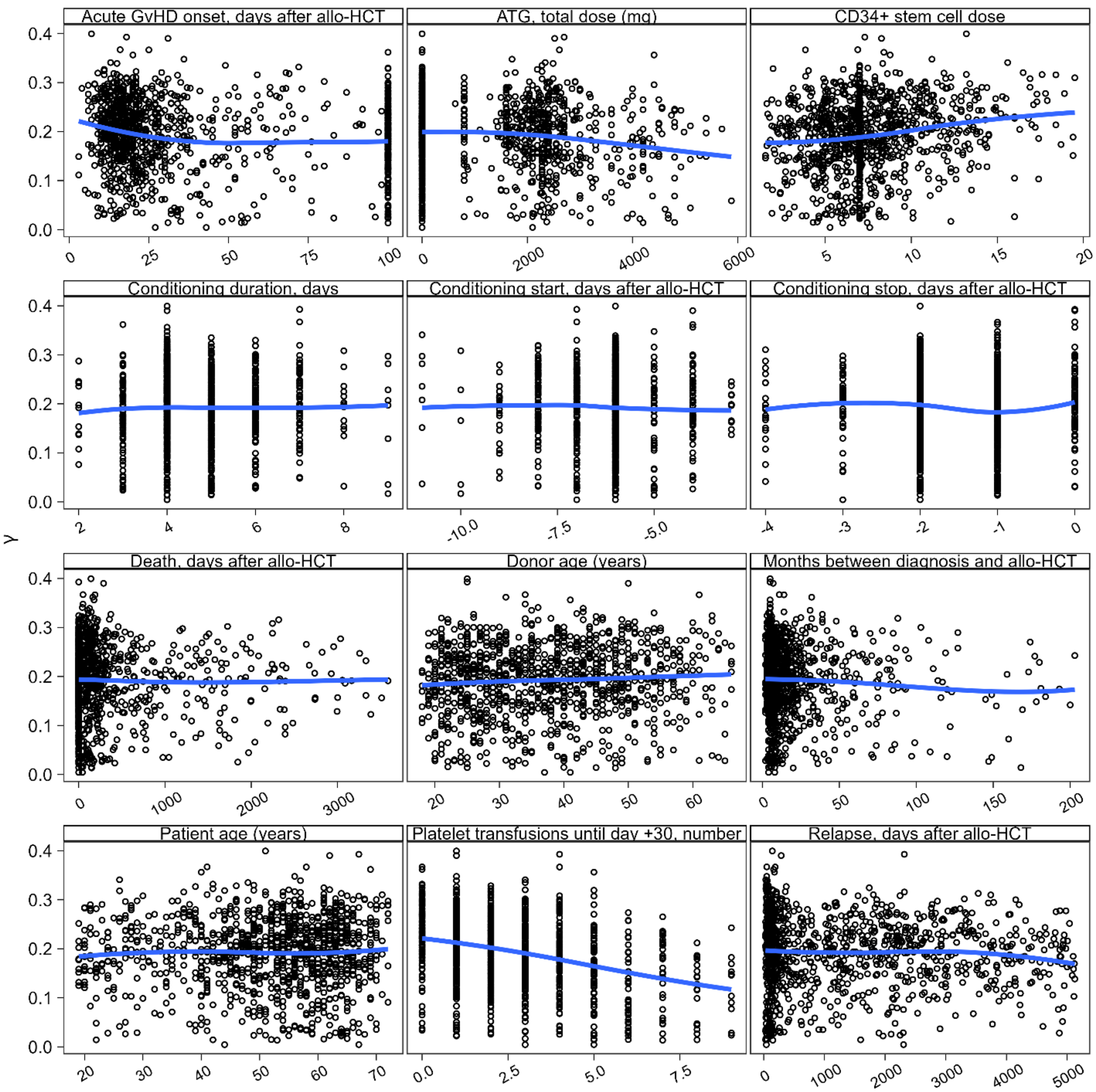

Figure S7. Scatterplots of empirical Bayesian estimates of the feedback parameter Gamma and continuous covariates that were available for more than 80% of the patients in the retrospective cohort. The blue lines represent locally estimated scatterplot smoothing (LOESS) regressions. Abbreviations: ATG, anti-thymocyte globulin; GvHD, graft versus host disease.

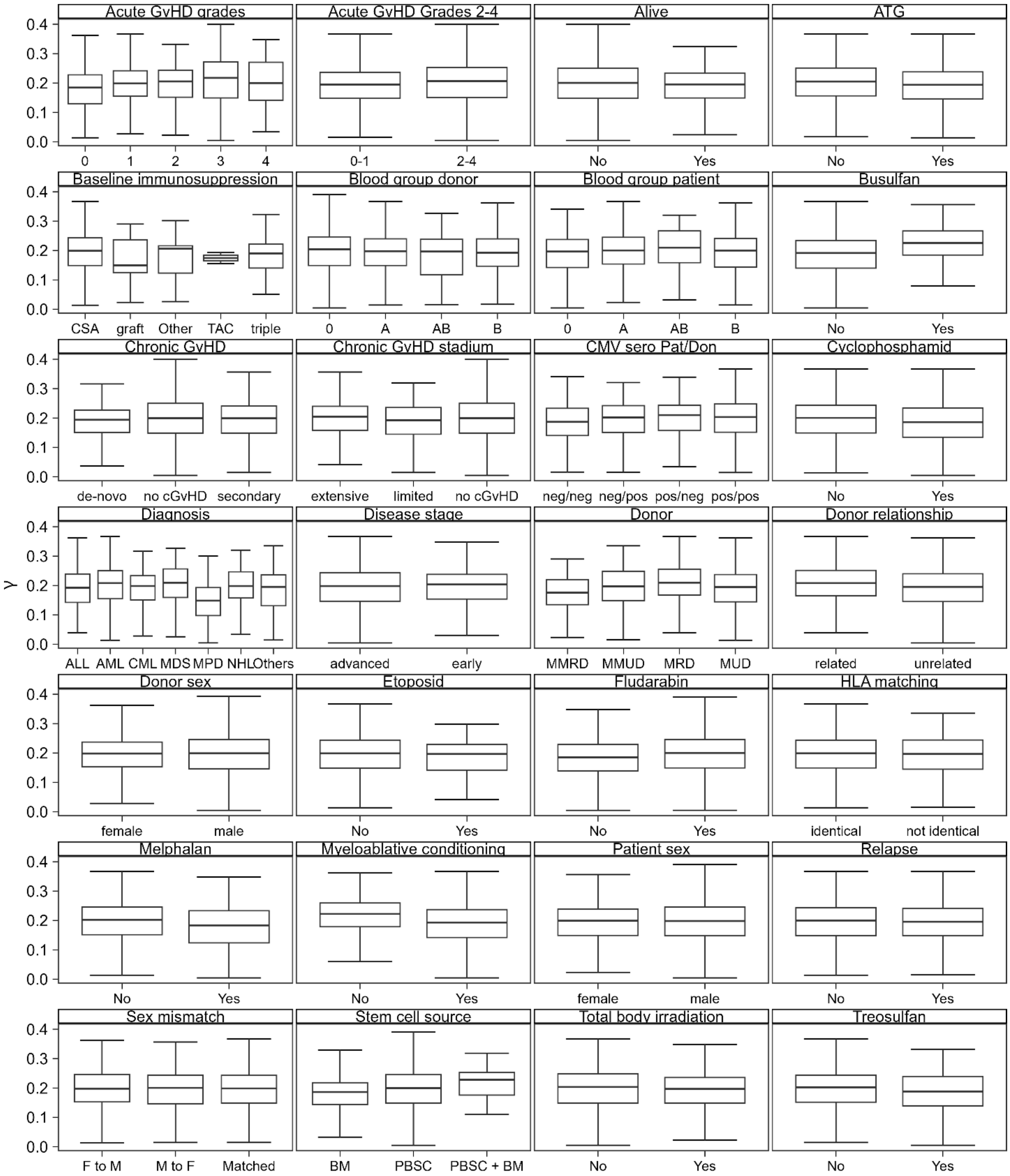

Figure S8. Box-and-whisker plots of empirical Bayesian estimates of the feedback parameter Gamma and categorical covariates that were available for more than 80% of the patients in the retrospective cohort. Abbreviations: ALL, Acute lymphoblastic leukemia; AML, Acute myeloid leukemia; ATG, anti-thymocyte globulin; BM, Bone marrow; CML, Chronic myeloid leukemia; CMV, cytomegalovirus; CSA, cyclosporine A; Don, stem cell donor; F, female; GvHD, graft versus host disease; HLA, human leukocyte antigen; neg, negative; M, male; MDS, Myelodysplastic syndromes; MMRD, Mis-matched related donor; MMUD, Mis-matched unrelated donor; MPD; Myeloproliferative Disorder; MRD, Matched related donor; MUD, matched unrelated donor; NHL, Non-Hodgkin's lymphoma; Pat, patient; PBSC, peripheral blood stem cell; pos, positive; TAC, tacrolimus.

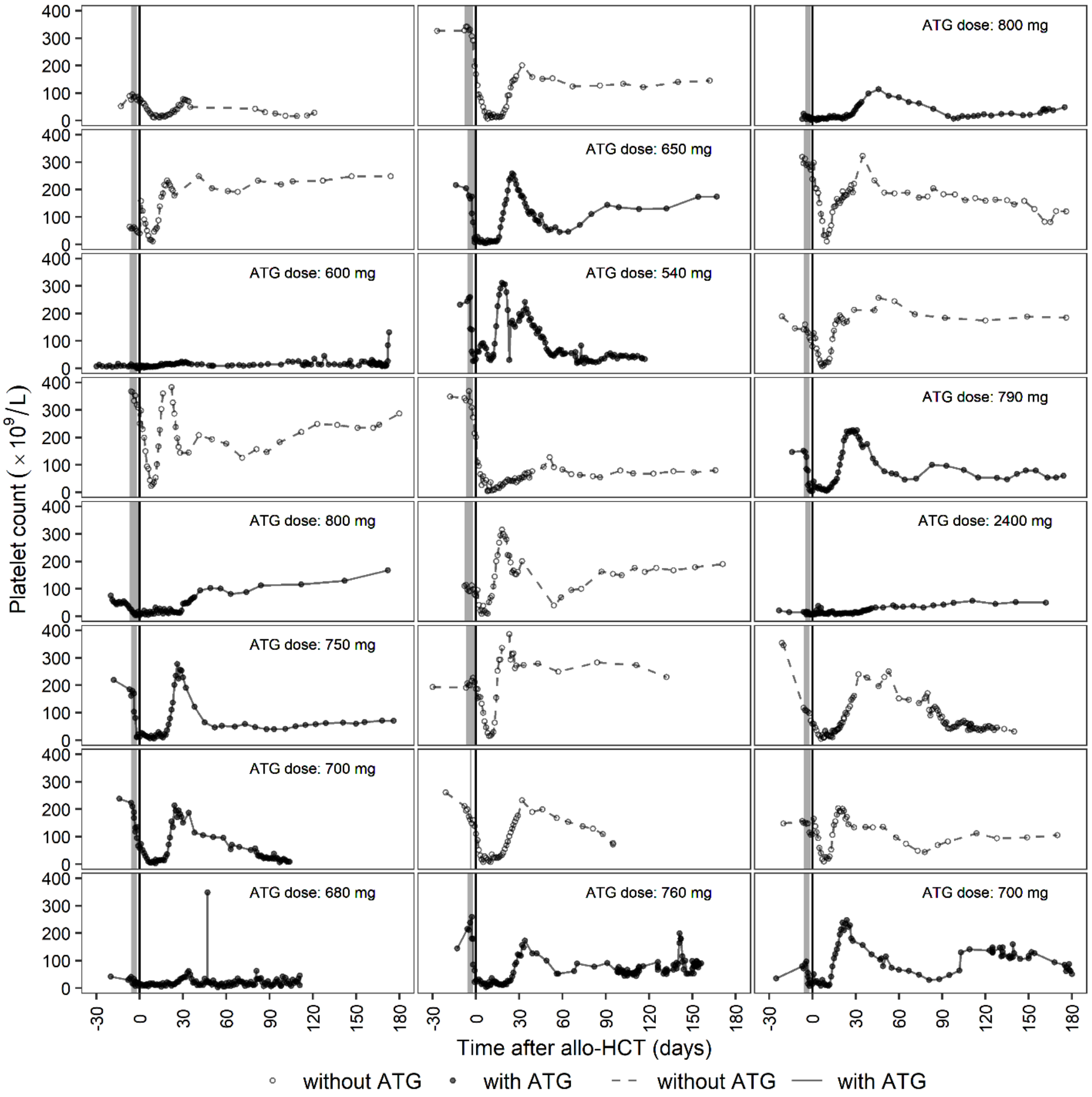

Figure S9. Randomly selected observed platelet–time profiles of 24 patients with a minimum observation period of 80 days post-HCT from the training dataset are shown. The grey background represents the individual period of conditioning. The circles (dashed lines) and dots (solid lines) present the observed platelet counts of patients without and with ATG treatment, respectively. ATG, anti-thymocyte globulin.

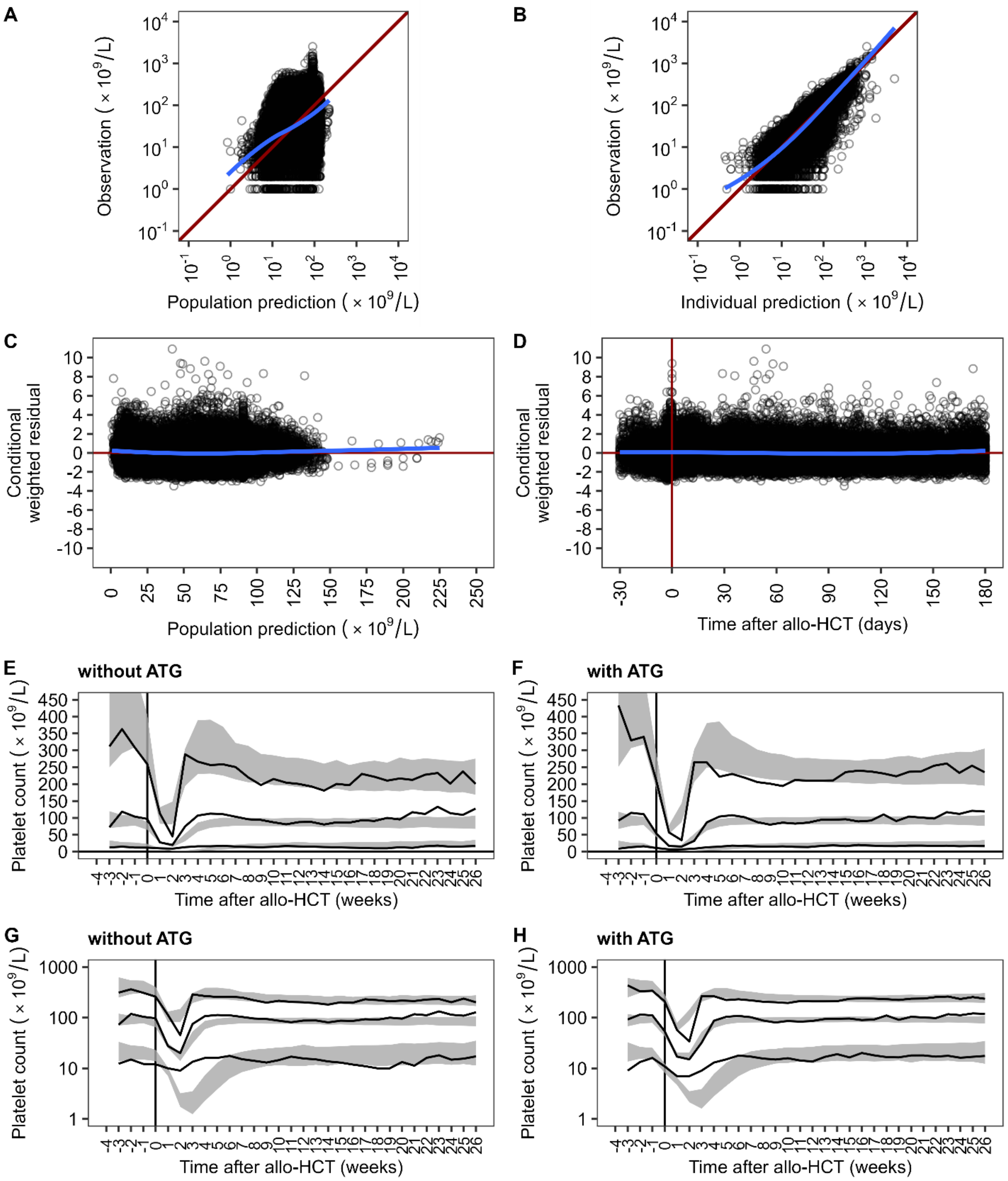

Figure S10. Goodness-of-fit plots for the final model. (**A, B**) Observed versus population predicted (**A**) and individual predicted (**B**) platelet counts. Solid lines indicate lines of identity. (**C, D**) Conditional weighted residuals versus population prediction (**C**) and time after allo-HCT (**D**). (**A–D**) The blue lines represent smooth regression curves resulting from locally estimated scatterplot smoothing (LOESS) regression. (**E–H**) The final model was evaluated using visual predictive check (VPC) based on 500 replicates of the test dataset, which is presented on a linear (**E, F**) and semi-log (**G, H**) scale. The grey shaded areas show the 95% confidence interval for the model predicted median, 5th and 95th percentiles without (**E, G**) and with (**F, H**) ATG treatment. The solid lines present the median, 5th and 95th percentiles of observed platelet counts without (**E**) and with (**F**) ATG treatment. ATG, anti-thymocyte globulin.

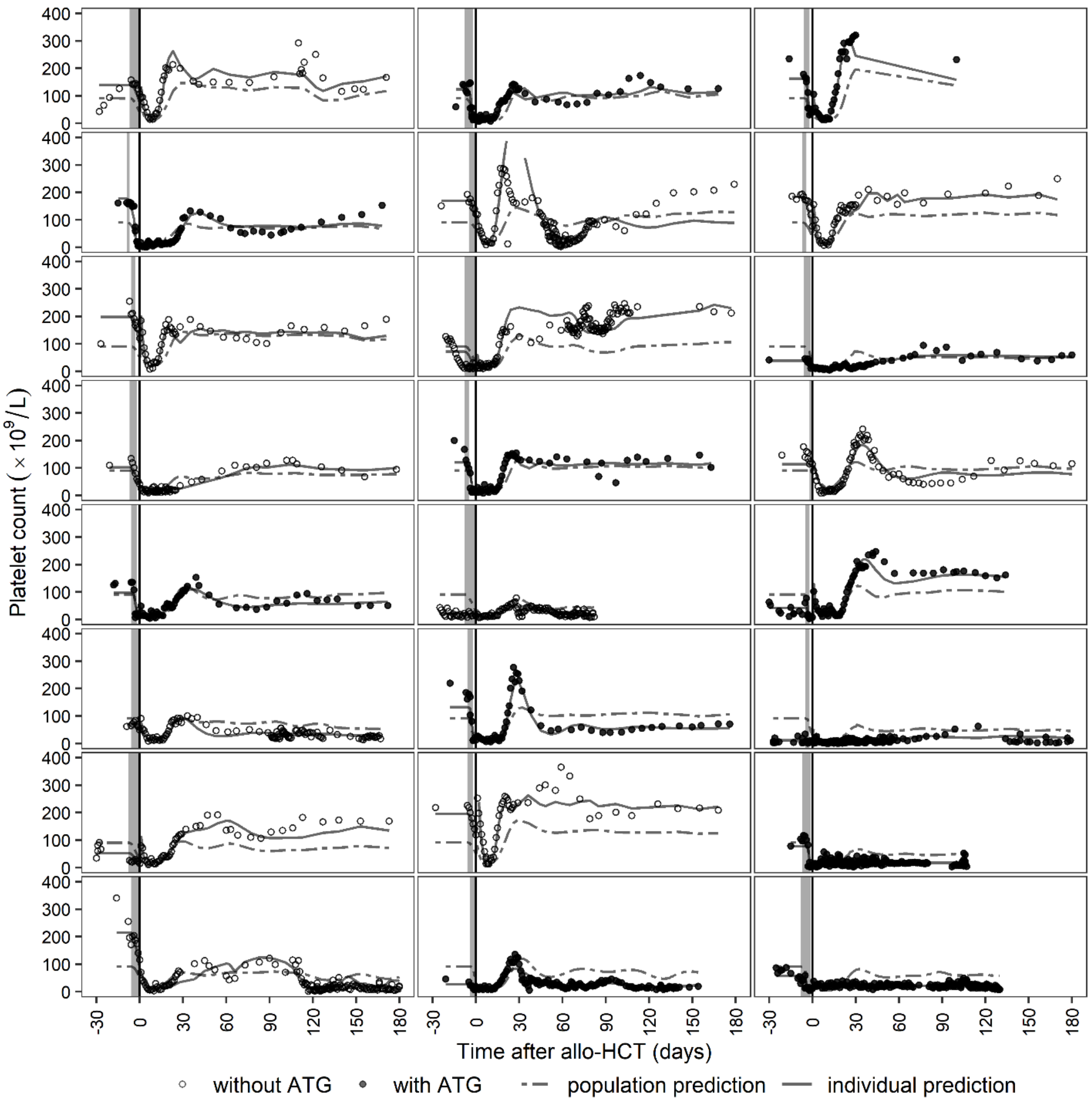

Figure S11. Randomly selected model predicted platelet–time profiles of 24 patients with a minimum observation period of 80 days post-HCT from the training dataset are shown. The grey background represents the individual period of conditioning. The black circles and dots present the observed platelet counts of patients without and with ATG treatment, respectively. The solid lines present the individual prediction of the model, and the dashed lines present the population prediction of the model. ATG, anti-thymocyte globulin.

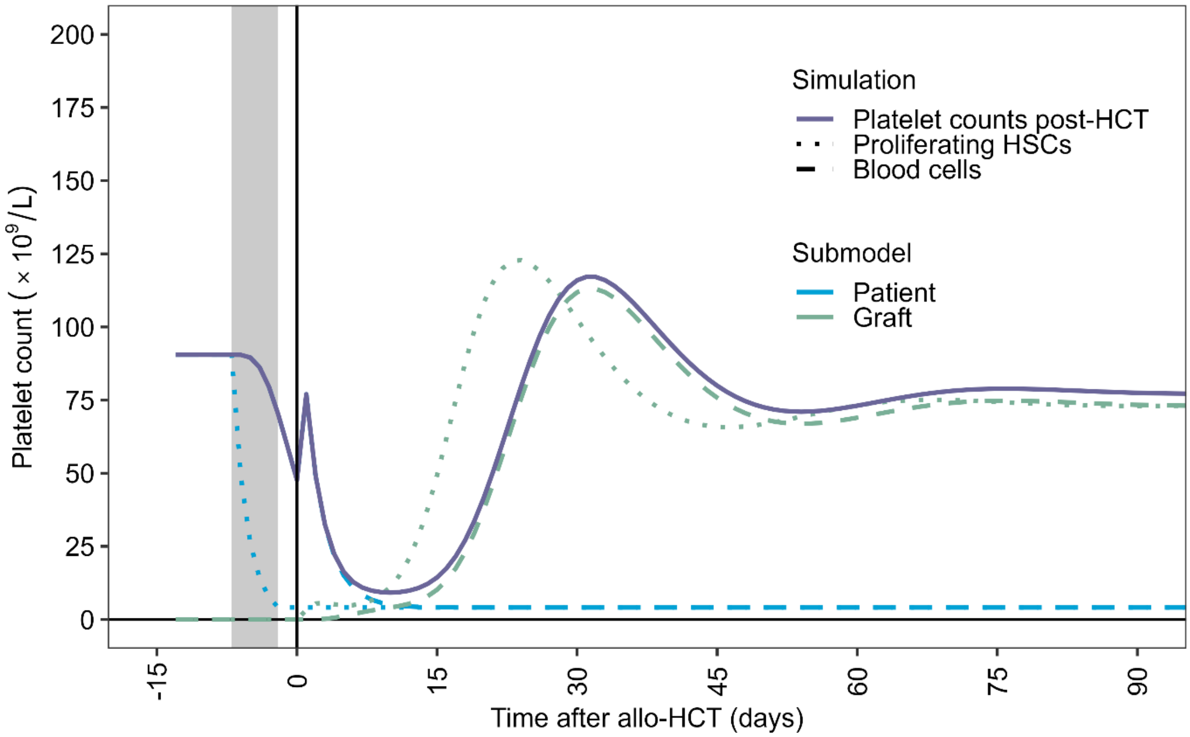

Figure S12. Simulation of a platelet count-time profile for a typical patient after allo-HCT including the simulated patient- and donor-derived hematopoietic stem cells and blood cells which derive from the patient and the graft. The purple solid line presents the simulated platelet counts of a typical patient, which is derived from the sum of blood cells from the patient and the graft model. The dotted and dashed lines show submodel-specific proliferating HSCs and peripheral blood cells, respectively. The blue and green lines indicate cells originating from the patient and the graft model, respectively. Abbreviations: HSC, hematopoietic stem cells.

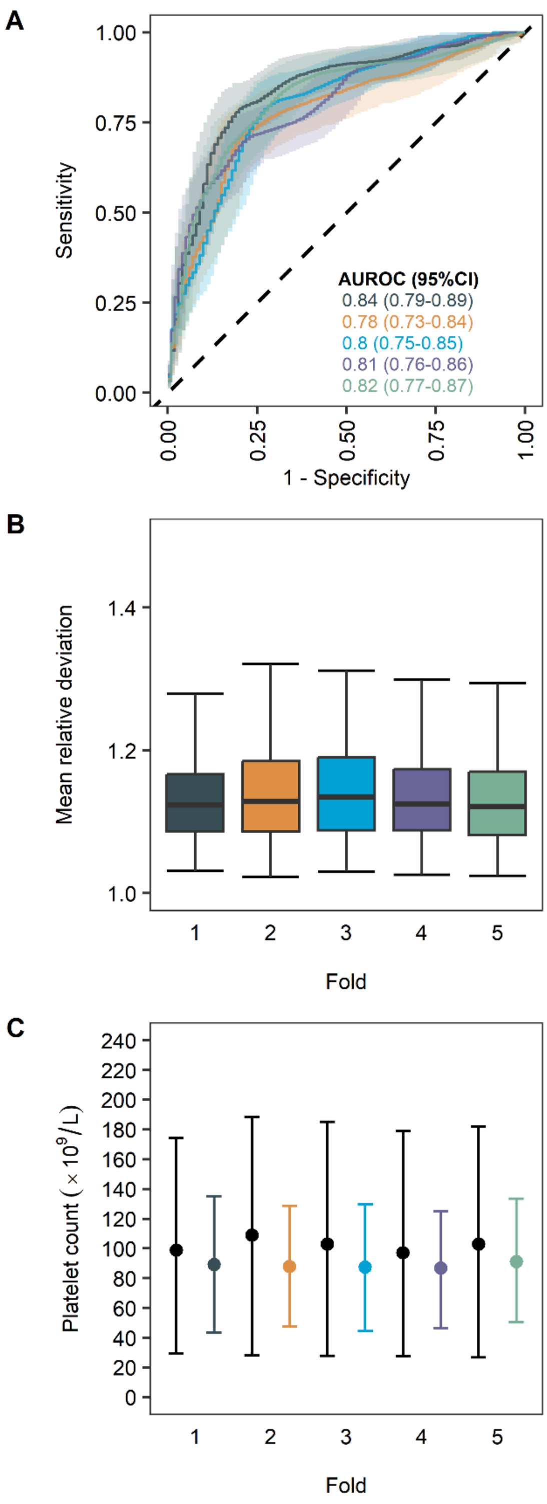

Figure S13. Predictive performance of the final model during model evaluation (five-fold cross-validation). (**A**) ROC curves and respective AUROC values. The shaded areas show the 95% confidence interval for AUROC values calculated from bootstrap analysis (n=1000). (**B**) Box-plots of mean relative deviations calculated from individual predicted platelet-time profiles. (**C**) Mean, 10th and 90th percentile values of all mean observed and all mean individual predicted platelet counts during the individual prediction period are shown. **A–C** Colours show the different folds. AUROC, area under the receiver-operating characteristic curve; ROC, receiver-operating characteristic.

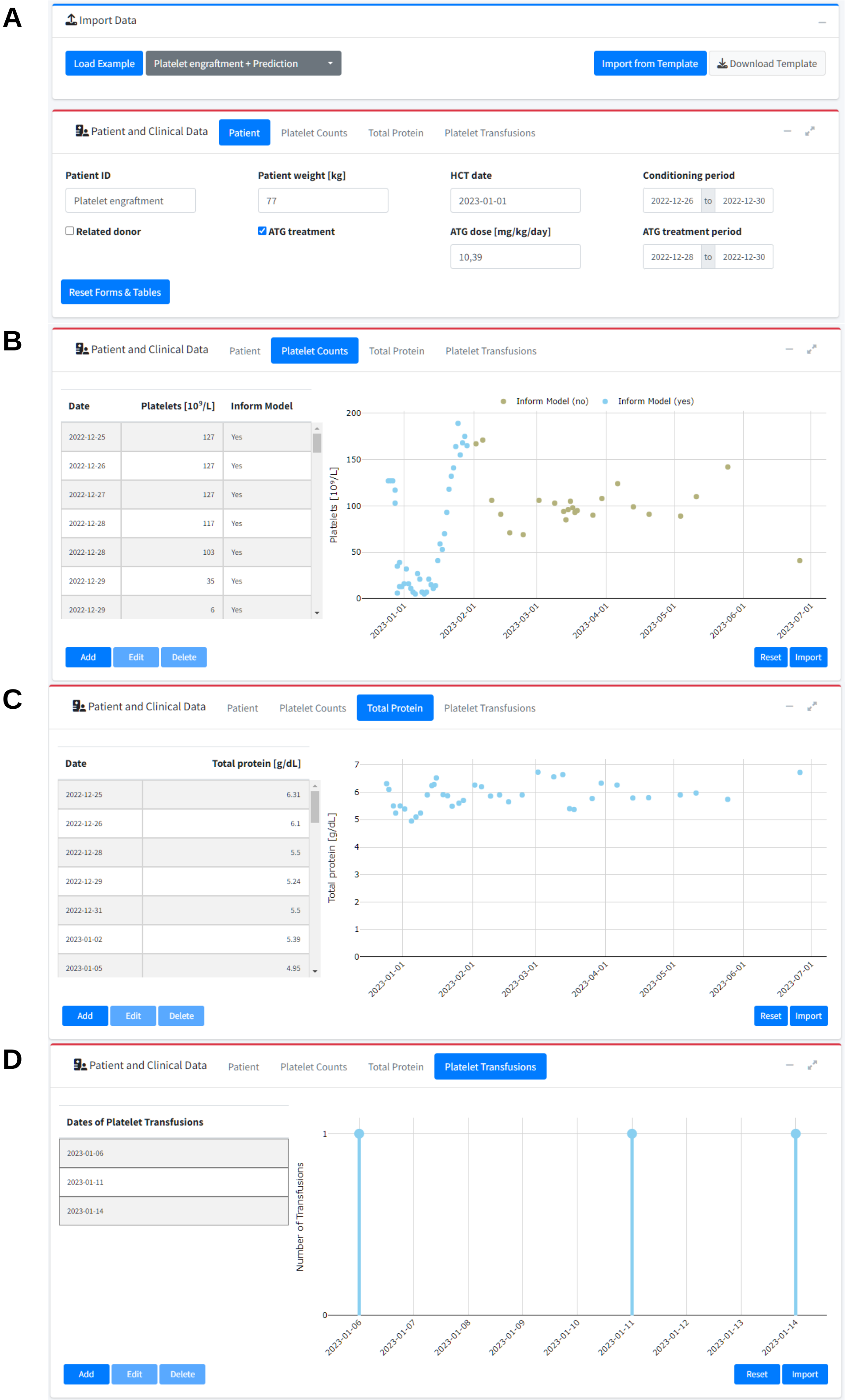

Figure S14. Screencaptures of the "Precise Platelets" application input panels. The web-based demonstrator application (https://hsct.precisiondosing.de) for the prediction of platelet counts up to 180 days post-HCT is driven by the presented model and allows the input of patient- and HCT-specific conditioning data (**A**), observed platelet counts (**B**) to inform the maximum a posteriory estimator (optional), measured total protein (**C**; optional) and information about platelet transfusions (**D**, optional).

A

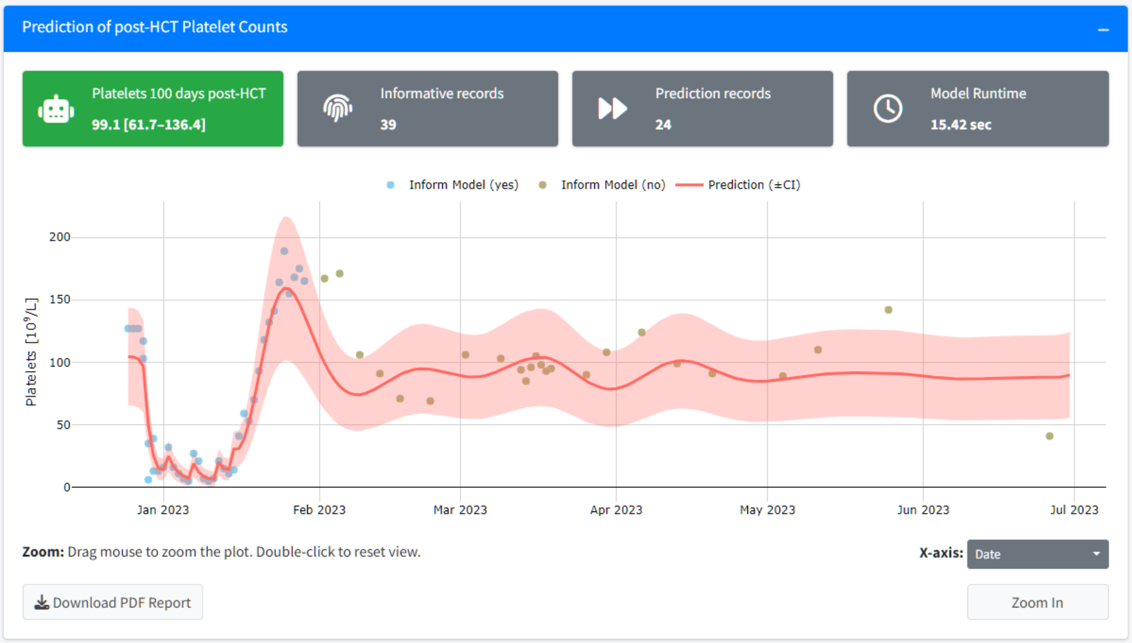

B

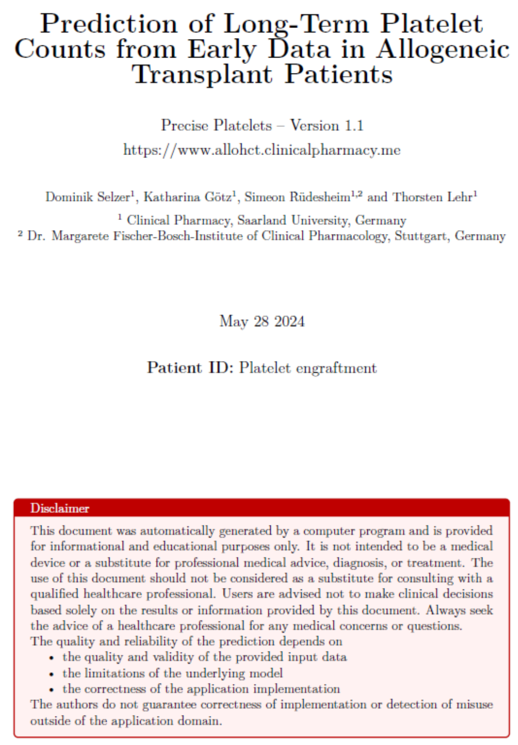

Prediction of Long-Term Platelet Counts from Early Data in Allogeneic Transplant Patients

Precise Platelets – Version 1.1

https://www.allohct.clinicalpharmacy.me

Dominik Selzer[1], Katharina Götz[1], Simeon Rüdesheim[1,2] and Thorsten Lehr[1]

[1] Clinical Pharmacy, Saarland University, Germany

[2] Dr. Margarete Fischer-Bosch-Institute of Clinical Pharmacology, Stuttgart, Germany

May 28 2024

Patient ID: Platelet engraftment

Disclaimer

This document was automatically generated by a computer program and is provided for informational and educational purposes only. It is not intended to be a medical device or a substitute for professional medical advice, diagnosis, or treatment. The use of this document should not be considered as a substitute for consulting with a qualified healthcare professional. Users are advised not to make clinical decisions based solely on the results or information provided by this document. Always seek the advice of a healthcare professional for any medical concerns or questions.
The quality and reliability of the prediction depends on

- the quality and validity of the provided input data
- the limitations of the underlying model
- the correctness of the application implementation

The authors do not guarantee correctness of implementation or detection of misuse outside of the application domain.

Figure S15. Screencaptures of the "Precise Platelets" application output. The web-based demonstrator application (https://hsct.precisiondosing.de) for the prediction of platelet counts up to 180 days post-HCT is driven by the presented model and predictions are interactively presented within the application (**A**) and via comprehensive PDF report with an exemplary cover sheet show (**B**).

NONMEM control stream (NM-TRAN) code

```
$SIZES NO=5000 PD=-120

$PROBLEM PD platelet reconstitution

$INPUT ID TIME AMT RATE EVID CMT DV MDV CONDTREAT ATG ATGDOSE DUR KINSHIP PROT
$DATA ..\DATASET\data.csv

$SUBROUTINES ADVAN13 TOL=5

$MODEL
COMP(STEM)            ;HSCs patient
COMP(TRN1)            ;Transit 1 patient
COMP(TRN2)            ;Transit 2 patient
COMP(TRN3)            ;Transit 3 patient
COMP(CIRC)            ;PLTs patient
COMP(effect)          ;HCT-effect
COMP(ATG)             ;ATG conc. (K-PD)
COMP(GRAFT)           ;Graft cells
COMP(GSTEM)           ;HSCs graft
COMP(GTRN1)           ;Transit 1 graft
COMP(GTRN2)           ;Transit 2 graft
COMP(GTRN3)           ;Transit 3 graft
COMP(GCIRC)           ;PLTs graft
COMP(GRAFT)           ;Transit Graft cells

$PK
;--------------------PD-----------------------------------
BASE1 = THETA(1)*EXP(ETA(1))                          ;Baseline platelet (PLT) count pre-HCT

A_0(1)=BASE1
A_0(2)=BASE1
A_0(3)=BASE1
A_0(4)=BASE1
A_0(5)=BASE1
A_0(6)=0
A_0(7)=0
A_0(8)=0
A_0(9)=0
A_0(10)=0
A_0(11)=0
A_0(12)=0
A_0(13)=0
A_0(14)=0

TVMMT = THETA(2)
MMT = TVMMT                                           ;Mean maturation time pre-HCT
KPAT = 4/MMT

DRUG = 0
IF (CONDTREAT.EQ.1) DRUG = THETA(3)*EXP(ETA(2))      ;Inhibition effect of conditioning treatment

SHPGA = THETA(14)
ETAGA = (EXP(ETA(6))**SHPGA-1)/SHPGA                  ;Box-Cox transformation of ETA distribution
TVGA = THETA(4)
GA = TVGA*EXP(ETAGA)                                  ;Gamma, feedback mechanism parameter

D7 = DUR
F7 = ATGDOSE                                          ;ATG, individual daily dose amount in mg

effect = THETA(7)                                     ;Prohibition effect of ATG
KINT = THETA(8)                                       ;ATG kinetics, elimination rate constant

KNSHP = 1                                             ;Define donor relation (1=related, 0=unrelated)
IF (KINSHIP.GT.1.5) KNSHP = 0                         ;Dataset: Kinship 1=related,2=unrelated

D5 = DUR
PEAK = THETA(9)*(1 + KNSHP*THETA(17))
F5 = PEAK*EXP(ETA(7))                                 ;Apheresis cells, incr. in PLT counts on day +1
IF (TIME.GT.0) F5 = THETA(12)*EXP(ETA(8))             ;PLT concentrates, incr. in PLT counts next day
```

```
COVATG = 0
IF (ATG.NE.0) COVATG = 1                            ;ATG treatment

D6 = DUR
TVF6 = THETA(10)
F6 = TVF6*EXP(ETA(5))                               ;HCT-effect
KEL = THETA(11)               ;HCT-effect elimination rate constant

TVGMMT = THETA(6)
GMMT = TVGMMT*EXP(ETA(4))                           ;Mean maturation time post-HCT
KGT = 4/GMMT

D8 = DUR
SHPF8 = THETA(15)
ETAF8 = (EXP(ETA(3))**SHPF8-1)/SHPF8                ;Box-Cox transformation of ETA distribution
TVF8 = THETA(5)
F8 = TVF8*EXP(ETAF8)                                ;Graft cells, baseline PLT count post-HCT

GRAFTCELLS = F8

TTBM = THETA(13)                                    ;Time to bone marrow
KGR = 2/TTBM

COVT = THETA(16)                                    ;Total protein parameter for HSC proliferation


$DES
FBM = 1                                             ;Feedback mechanism from blood cells to HSCs
IF (T.GT.0) FBM = (GRAFTCELLS/(A(13)+0.0001))**(GA)

COVTIME = ((PROT / 5.43)**COVT)                     ;Continuous effects of total protein (PROT)
                                                    ;on the proliferation of donor-derived HSCs

DADT(1) = KPAT*A(1)*(1 - DRUG)-KPAT*A(1)
DADT(2) = KPAT*A(1)-KPAT*A(2)
DADT(3) = KPAT*A(2)-KPAT*A(3)
DADT(4) = KPAT*A(3)-KPAT*A(4)
DADT(5) = KPAT*A(4)-KPAT*A(5)*(1+effect*A(7))
DADT(6) = -KEL*A(6)
DADT(7) = -KINT*A(7)
DADT(8) = -KGR*A(8)
DADT(9) = KGT*A(9)*FBM*(1 - A(6))*COVTIME-KGT*A(9)+KGR*A(14)
DADT(10) = KGT*A(9)-KGT*A(10)
DADT(11) = KGT*A(10)-KGT*A(11)
DADT(12) = KGT*A(11)-KGT*A(12)
DADT(13) = KGT*A(12)-KGT*A(13)
DADT(14) = KGR*A(8)- KGR*A(14)


$ERROR
STEMS = A(1)
TRNS1 = A(2)
TRNS2 = A(3)
TRNS3 = A(4)
CIRC = A(5)
HCT = A(6)
ATGPK = A(7)
GRAFT = A(8)
GSTEMS = A(9)
GTRNS1 = A(10)
GTRNS2 = A(11)
GTRNS3 = A(12)
GCIRC = A(13)

IPRED = A(5)+A(13)
DEL = 0
IF (IPRED.EQ.0) DEL = 0.0001

W = IPRED
IRES = DV-IPRED
IWRES = IRES/(W+DEL)
Y = IPRED + W*EPS(1) + EPS(2)
```

```
$THETA
(0, 90.5)       ;1 Baseline platelet count pre-HCT
(0, 6.11)       ;2 Mean maturation time pre-HCT
(0, 0.945)      ;3 Inhibition effect of the conditioning treatment
(0, 0.192)      ;4 Gamma, Feedback mechanism parameter
(0, 72.7)       ;5 Graft cells
(0, 6.99)       ;6 Mean maturation time post-HCT
(0, 0.00212)    ;7 Prohibition effect of ATG
(0, 0.693)      ;8 KINT, ATG elimination rate constant
(0, 20.1)       ;9 Apheresis cells, increase in platelet counts on day +1
(0, 4.22)       ;10 HCT-effect
(0, 0.353)      ;11 KEL, HCT-effect elimination rate constant
(0, 10.6)       ;12 Platelet concentrates
(0, 1.54)       ;13 TTBM, Time to bone marrow
(-1.28)         ;14 SHPGA, ETA distribution shape for γ
(-0.484)        ;15 SHPF8, ETA distribution shape for Graft cells
(0.197)         ;16 Total protein parameter for HSC proliferation
(2.75)          ;17 Graft relation parameter for Apheresis cells

$OMEGA BLOCK(2)
0.843           ;1 IIV_BASE
0.589 1.2       ;2 IIV_DRUG

$OMEGA BLOCK(2)
0.523           ;3 IIV_GRAFTCELLS
-0.291 0.602    ;4 IIV_GMMT

$OMEGA 0.565    ;5 IIV_HCT
$OMEGA 0.213    ;6 IIV_GA
$OMEGA 2.24     ;7 IIV_apheresis cells
$OMEGA 0.379    ;8 IIV_PLT concentrates

$SIGMA 0.112    ;PD_prop
$SIGMA 15.8     ;PD_add

$EST METHOD=SAEM INTERACTION PRINT=100 NBURN=1000 NITER=1000 ISAMPLE=1000
$EST METHOD=IMP EONLY=1 ISAMPLE=1000 NITER=10 PRINT=1 MAPITER = 0
$COV UNCONDITIONAL

$TABLE ID TIME DV IPRED EVID MDV CMT NOPRINT FILE=sdtab01085
```
